# A Bayesian Attractor Model for Perceptual Decision Making

**DOI:** 10.1371/journal.pcbi.1004442

**Published:** 2015-08-12

**Authors:** Sebastian Bitzer, Jelle Bruineberg, Stefan J. Kiebel

**Affiliations:** 1 Max Planck Institute for Human Cognitive and Brain Sciences, Department of Neurology, 04103 Leipzig, Germany; 2 Department of Psychology, Technische Universität Dresden, 01062 Dresden, Germany; 3 Department of Philosophy, Institute for Logic, Language and Computation, University of Amsterdam, 1012 GC, Amsterdam, the Netherlands; 4 Biomagnetic Centre, Hans Berger Clinic for Neurology, University Hospital Jena, 07747 Jena, Germany; Indiana University, UNITED STATES

## Abstract

Even for simple perceptual decisions, the mechanisms that the brain employs are still under debate. Although current consensus states that the brain accumulates evidence extracted from noisy sensory information, open questions remain about how this simple model relates to other perceptual phenomena such as flexibility in decisions, decision-dependent modulation of sensory gain, or confidence about a decision. We propose a novel approach of how perceptual decisions are made by combining two influential formalisms into a new model. Specifically, we embed an attractor model of decision making into a probabilistic framework that models decision making as Bayesian inference. We show that the new model can explain decision making behaviour by fitting it to experimental data. In addition, the new model combines for the first time three important features: First, the model can update decisions in response to switches in the underlying stimulus. Second, the probabilistic formulation accounts for top-down effects that may explain recent experimental findings of decision-related gain modulation of sensory neurons. Finally, the model computes an explicit measure of confidence which we relate to recent experimental evidence for confidence computations in perceptual decision tasks.

## Author Summary

How do we decide whether a traffic light signals stop or go? Perceptual decision making research investigates how the brain can make these simple but fundamentally important decisions. Current consensus states that the brain solves this task simply by accumulating sensory information over time to make a decision once enough information has been collected. However, there are important, open questions on how exactly this accumulation mechanism operates. For example, recent experimental evidence suggests that the sensory processing receives feedback about the ongoing decision making while standard models typically do not assume such feedback. It is also an open question how people compute their confidence about their decisions. Furthermore, current decision making models usually consider only a single decision and stop modelling once this decision has been made. However, in our natural environment, people change their decisions, for example when a traffic light changes from green to red. Here, we show that one can explain these three aspects of decision making by combining two already existing modelling techniques. This resulting new model can be used to derive novel testable predictions of how the brain makes perceptual decisions.

## Introduction

Research in perceptual decision making investigates how people categorise observed stimuli. By presenting stimuli embedded in large amounts of noise, experimenters prolong the time it takes a subject to make a decision about the stimulus. This makes the decision making process observable for hundreds of milliseconds and enables experiments about the underlying mechanisms [1]. For example, in the well-known random dot motion task subjects typically have to categorise a cloud of moving dots according to whether it moves in one of two opposing directions [2–4]. By decreasing the fraction of coherently moving dots the task is made more difficult such that subjects respond slower and make more errors.

Such increases in reaction time for more difficult categorisations motivated models that describe decision making as an accumulation of noisy evidence towards a bound [5, 6]. One of the key findings is that such bounded accumulation models fit accuracy and reaction time distributions of decision makers well [6–8]. Furthermore, electrophysiological research has found support for an accumulation mechanism: neurons in different areas of monkey brains exhibit steadily increasing firing rates dependent on stimulus reliability, e.g. [1, 9–12]. In humans, correlates of evidence accumulation have been found with functional magnetic resonance imaging [13, 14] and magneto-/electroencephalography [15–19].

The two best-known models of perceptual decision making are drift-diffusion and attractor models. Drift-diffusion models implement accumulation to a bound using diffusion processes [7, 20–22] and can be understood in terms of statistically optimal sequential decision making [20]. Bayesian models of perceptual decisions provide a direct link between the computation of evidence from the sensory input and the statistically optimal accumulation of this evidence [23–25]. In contrast, attractor models were developed as neurophysiologically inspired spiking-neuron models of perceptual decision making [26], but can also be described by simpler firing rate models [27, 28]. Attractor models use winner-take-all dynamics to implement accumulation which is nonlinear over time. This nonlinear accumulation is the key difference to drift-diffusion models, which are based on linear accumulation. Both types of models seem to make mostly the same predictions [29, 30], yet exhibit subtle differences in favour of attractor models when considering experimental evidence [31–33] but see [34].

Bayesian inference provides an optimal approach for combining noisy sensory evidence with internal dynamics and seems generally useful as a basic mechanistic principle for perceptual decision making. For example, drift-diffusion models are strongly connected to Bayesian models of perceptual decision making [23–25]. Therefore, the question arises what exactly a Bayesian inference approach would have to offer for attractor models. Here, we address this question by combining a variant of the nonlinear attractor model with Bayesian inference. The resulting new model, which we call the Bayesian attractor model (BAttM), combines the neurophysiological motivation of the attractor model with the explicit evidence computation formalism of the Bayesian machinery. As we will show, the BAttM is a quantitative model and fits well to behavioural data (reaction times and choice). Furthermore, we will highlight three key advantages of the BAttM that go beyond the standard features of both attractor and drift-diffusion models.

First, the BAttM naturally models changes in decisions that reflect changes in the underlying category. Such changes of an already made decision are an important part of our environment, e.g., a switching traffic-light, but have not been considered by previous models. Rather, drift-diffusion [35] and attractor models [26, 31] have been adapted to model ‘changes of mind’ which are different from ‘re-decisions’ considered here (for more details on the difference see Discussion).

Second, the BAttM provides a natural explanation for top-down modulation of the sensory gain that controls evidence extraction during the decision making process. Such gain modulation has been implicated in attentional phenomena such as found in feature-based attention [36–38]. In addition, early sensory neurons have been found to exhibit within-trial gain modulation that appears to depend on the final choice in a trial [39, 40]. The BattM explains these phenomena in terms of a state-dependent, top-down gain mechanism which is absent from both drift-diffusion and attractor models.

Third, the BAttM provides an explicit measure of confidence that reproduces the experimentally established dependence of confidence ratings on decision outcome and task difficulty [41–43]. In particular and in contrast to both drift-diffusion and attractor models, the probabilistic formulation of the BAttM yields a quantitative measure of confidence that reflects the decision maker’s internal expectations and provides a meaningful quantitative interpretation of the bound.

## Models

The BAttM consists of four major components: i) an abstract model of the experimental stimuli used as input to the decision process of a decision maker, ii) a generative model of the stimuli implementing expectations of the decision maker, iii) a Bayesian inference formalism and iv) a decision criterion, see also [23]. In the following, we define these components in turn and, particularly, clarify the role of attractor dynamics in the model and how this differs from previously suggested attractor models of perceptual decision making. We start by formalising a notion of attractor models.

### Pure attractor models

Attractor models of perceptual decision making were originally proposed as neurophysiologically plausible implementation of noisy decision making [26]. In particular, [26] introduced a spiking neuron network which implements decisions through an attractor dynamics based on two mutually inhibiting pools of neurons. By using a mean-field approach this model has been reduced to a relatively small set of differential equations [28], see also [27, 32].

Apart from the neurobiological motivation, attractor models mainly differ from prevalent diffusion models of decision making by the nonlinear accumulation of evidence: The mutual inhibition between alternatives leads to faster accumulation for an alternative as more evidence for that alternative is accumulated, that is, decisions for an alternative are attracting. In the present work we capture this decisive property of attractor models with a simpler, more abstract Hopfield network [44]. The Hopfield dynamics describes how state variables *z*_*i*_ (the activities of units in the Hopfield network) evolve through time. Each state variable corresponds to one decision alternative. Intuitively, large values of state variable *z*_*i*_ indicate large amounts of evidence for decision alternative *i*. The Hopfield dynamics implements lateral inhibition between and self-excitation of state variables. As a result, it exhibits winner-takes-all dynamics which ensures stable and unambiguous decision making between alternatives. In particular, the Hopfield dynamics has stable fixed points *ϕ*_*i*_, each identifying one decision alternative *i*. For further details see Methods.

**Fig 1:**
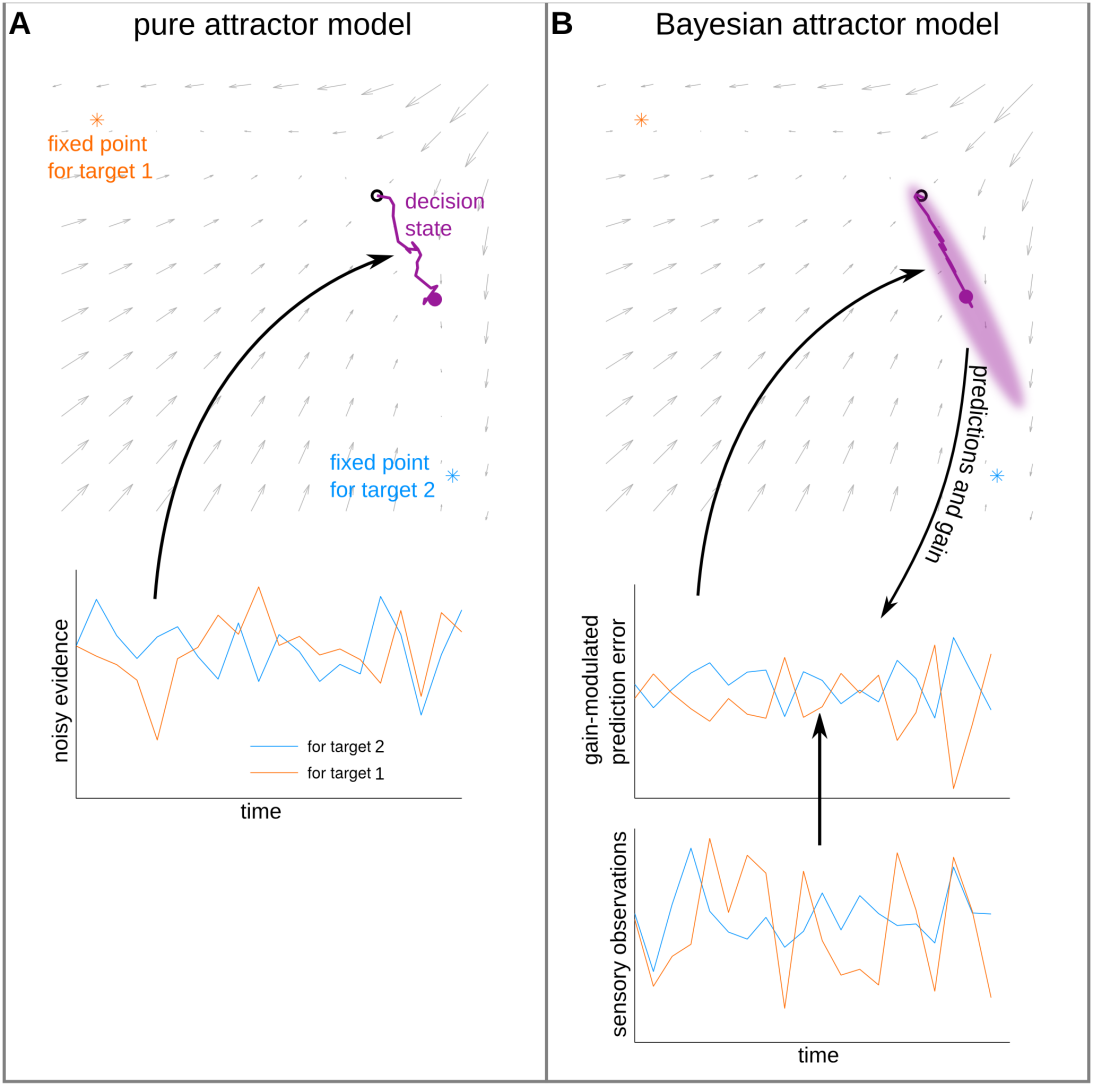
Schematic comparing a pure attractor model of decision making with the Bayesian attractor model. Both plots show illustrative snapshots of the two evolving decision states while in transit towards a fixed point where a decision will be made. (A) In a pure attractor model, on the way to a fixed point, the decision state (violet) is evolving according to attractor dynamics (grey arrows). From an initial, unstable fixed point (empty, black circle) the decision state is driven by noisy evidence into one of two attracting, stable fixed points, each of which correspond to a decision alternative. (B) In the Bayesian attractor model the same attractor dynamics is used as generative model for sensory observations. The decision state effects, in a top-down fashion, both internal predictions and gain. These are in turn used together with sensory observations to compute gain-modulated prediction errors which drive updates of the decision state. The model represents uncertainty over the decision state (shaded, violet ellipse) and allows to define the decision criterion directly in terms of confidence in the decision. We show in Results that this recurrent principle stabilises the location of fixed points of the attractor dynamics while at the same time maintaining the ability to reliably switch decisions after a change in stimulus.

By abstracting from details of the particular attractor dynamics used in different models, previous attractor models of decision making may be formalised (in discretised form) as

$$z_{t}-z_{t-\Delta t}=\Delta tf\left(z_{t-\Delta t}\right)+I_{t}$$ (1)

where *f*(**z**) is a function defining an attractor dynamics for the vector of state variables **z**, which we also call decision state (cf. Table 1). The external input **I**_*t*_ varies with stimulus strength, includes noise, directly drives the attractor dynamics and reflects momentary evidence in decision making (see Fig 1A). Typically, when one of the state variables *z*_*i*_ reaches a certain threshold, the model indicates a decision for the corresponding alternative *i*.

**Table 1:** Key variables and parameters of the BAttM. These variables are defined mathematically in the models section below.

**Variable**	**Name**	**Interpretation**
**z**	*decision state*	current state of attractor dynamics; consisting of state variables *z*_*i*_; one for each decision alternative
*s*	*noise level*	the actual amount of noise with which sensory observations are corrupted
*r*	*sensory uncertainty*	the amount of noise on sensory observations that the decision maker expects
*q*	*dynamics uncertainty*	the amount of noise with which the decision maker expects its decision state to be corrupted in each time step; the higher this uncertainty, the easier it is for the decision maker to switch between alternatives

We refer to models of this type as ‘*pure* attractor models’ which include the attractor models described above [26–28]. Note that pure attractor models are not informed about the stimulus itself or its features. Rather, they presume that their noisy input carries some information about a stimulus which is interpreted as evidence for or against the considered alternatives. Therefore, these models implicitly postulate that evidence for a decision is extracted by lower level sensory processes which are independent of the state of an ongoing decision. Under this assumption, pure attractor models cannot exhibit top-down gain control as a mechanism, because the decision state cannot provide feedback to the lower sensory level, see Fig 1A.

### Input model

Bayesian models infer the state of an unobserved variable (here the identity of a stimulus) from realisations of an observed variable [24, 45–47]. Here, we define these ‘observations’ and motivate them as feature representations in the brain.

Even though the BAttM can model tasks with multiple alternatives, we here focus on two-alternative forced choice tasks, as most commonly employed when investigating perceptual decisions. For example, in typical random dot motion (RDM) tasks subjects have to judge into which of two opposing directions a randomly moving cloud of dots moves on average [2–4]. By varying the percentage of coherently moving dots the task difficulty can be controlled.

We assume that the brain translates low-level sensory information, such as moving patters of light and dark spots on the retina, into stimulus feature vectors that are relevant for the current decision. In the RDM task a suitable feature may be the dominant motion direction in the stimulus, or a distribution over it. As the motion in the stimulus becomes less coherent, the dominant motion direction becomes more noisy.

The precise feature representation that the brain uses when making decisions, including the particular distribution of feature vectors, is largely unknown. Consequently, we take a suitably parsimonious approach and model (abstract) feature vectors as samples from one of two Gaussian distributions which represent the two alternatives in the decision task. In particular, a feature vector at time *t* is **x**_*t*_ ∼ 𝓝(***μ***_*i*_, *s* ^2^ **I**) where *s* is the standard deviation of the noise, or noise level (cf. Table 1) and ***μ***_*i*_ is the feature vector that would result, if alternative *i* was presented without noise. We set ***μ***_1_ = [0.71,0.71]^*T*^ (alternative 1) and ***μ***_2_ = [−0.71,−0.71]^*T*^ (alternative 2), that is, the feature vectors of the two alternatives occupy opposite positions on the unit circle.

**Fig 2:**
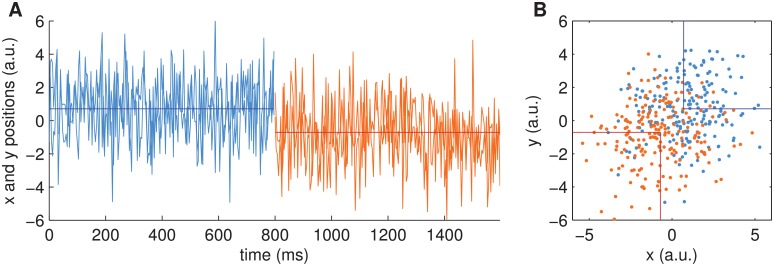
Example stimulus of single dot task, with a switch of target location. (A) The plot shows both x- and y- positions of the single dot throughout an example trial of 1600ms length. Every 40ms a new dot position is drawn. For 800ms positions are drawn from the first target (blue), i.e., a Gaussian with mean position [0.71, 0.71] (dark blue horizontal line) and standard deviation *s* = 2 in both dimensions. For the next 800ms positions are drawn from the second target (orange) around the mean [-0.71, -0.71] (red horizontal line) with the same standard deviation. (B) Same data as in (A), but plotted in 2D coordinates as when presented on a screen. Note that the observer would see only a single dot of neutral colour at any time throughout the trial and would have to decide whether the dot moves around the first (lower left) or second (upper right) target (indicated by lines).

This (feature) representation of the noisy stimulus has itself an interpretation as a perceptual decision making task. We use this interpretation here to illustrate the task that the brain, as decision maker, presumably solves when given noisy feature vectors as observations in a decision task: The feature vector **x** can be interpreted as the location of a single dot on a plane which moves randomly around one of two target positions. The single dot positions are sampled from an isotropic two-dimensional Gaussian with mean equal to one of the two targets. The task of the decision maker is to infer around which of the two target locations the single dot moves. Similarly to the RDM task, the difficulty of the task can be continuously varied by manipulating the ratio between the noise level and the distance between the two targets. In the two extremes, there is either no noise so that the correct target can be inferred easily, or the random movements are so large that one cannot infer the true target (i.e., the mean of the underlying Gaussian) with sufficient certainty. In Fig 2 we illustrate the dot movements across an example trial in this task.

### Generative model with attractor dynamics

The generative model of the decision maker implements its expectations about the incoming observations. More precisely, the generative model is a probabilistic model that defines the likelihood of observations under all possible hypotheses that the decision maker considers. Compared to pure attractor models the flow of information is reversed in the generative model: The generative model predicts a probability distribution over observations based on the current decision state and its winner-take all attractor dynamics. In contrast, in pure attractor models evidence extracted from the stimulus perturbs the decision state without any feedback from the decision state to the sensory evidence (cf. Fig 1).

A previous Bayesian model of perceptual decision making [23] defined independent generative models for the different alternatives in the decision task. The Bayesian attractor model complements the generative model with a competition between alternatives as implemented by attractor dynamics. In particular, the generative model defines a change in decision state from one time step to the next as

$$z_{t}-z_{t-\Delta t}=\Delta tf\left(z_{t-\Delta t}\right)+\sqrt{\Delta t}w_{t}$$ (2)

where *f*(**z**) is the Hopfield dynamics (Methods, Eq 9). **w**_*t*_ is a (Gaussian) noise variable with **w**_*t*_ ∼ 𝓝(**0**,**Q**) where **Q** = (*q* ^2^/Δ*t*)**I** is the isotropic covariance of the noise process and we call *q* ‘dynamics uncertainty’. It represents the (expected) state noise at the attractor level which can be interpreted as the propensity to switch between decisions (the higher the dynamics uncertainty, the more likely the state switches between the decision alternatives).

Given a decision state **z** the generative model predicts a probability distribution over observations by interpolating prototypical observations that represent the different alternatives:

x=Mσ(z)+v (3)

where **M** = [***μ***_1_,…, ***μ***_*N*_] contains the mean feature vectors defined in the input model above. This choice implements the reasonable assumption that the decision maker has learnt the average representations of the stimuli in feature space either through experience with the task, or from a suitable cue in the experiment. ***σ***(**z**) is the sigmoid-transformed decision state, that is, all state variables *z*_*j*_ are mapped to values between 0 and 1. Due to the winner-take-all mechanism of the Hopfield dynamics, its stable fixed points *ϕ*_*i*_ will map to vectors ***σ***(*ϕ*_*i*_) in which all entries are approximately 0 except for one entry which is approximately 1. Hence, the linear combination **M** ***σ***(**z**) associates each stable fixed point *ϕ*_*i*_ with feature vectors (observations) from one of the decision alternatives. When the Hopfield network is not in one of its stable fixed points, **M** ***σ***(**z**) interpolates between mean feature vectors ***μ***_*i*_ dependent on the sizes of individual state variables *z*_*j*_. Finally, **v** is a (Gaussian) noise variable with **v**_*t*_ ∼ 𝓝(**0**,**R**) where **R** = *r* ^2^ **I** is the expected isotropic covariance of the noise on the observations and we call *r* ‘sensory uncertainty’. It represents the *expected* noise level of the dot movement in the equivalent single dot decision task explained above (the higher the sensory uncertainty, the more noise is expected by the decision maker).

### Bayesian inference

By inverting the generative model using Bayesian inference we can model perceptual inference. Specifically, we use Bayesian online inference to infer the posterior distribution of the decision state **z**_*t*_, that is, the state of the attractor dynamics at time *t*, from sensory input, that is, all the sensory observations made up to that time point: **X**_Δ*t*:*t*_ = {**x**_Δ*t*_,…, **x**_*t*_}, given the generative model (Eqs 2, 3). The generative model postulates that the observations are governed by the Hopfield dynamics. Hence, the inference must account for the assumption that observations of consecutive time points depend on each other. In this case, inference over the decision state **z**_*t*_ is a so-called filtering problem which could be solved optimally using the well-known Kalman filter (see, e.g., [48]), if the generative model was linear. For nonlinear models, such as presented here, exact inference is not feasible. Therefore, we used the unscented Kalman filter (UKF) [49] to approximate the posterior distribution over the decision state **z**_*t*_ using Gaussians. Other approximations such as the extended Kalman filter [48], or sequential Monte Carlo methods [50] could also be used. We chose the UKF, because it provides a suitable tradeoff between the faithfulness of the approximation and computational efficiency.

The UKF is based on a deterministic sampling technique called the unscented transform [51][52], which provides a minimal set of sample points (sigma points). These sigma points are propagated through the nonlinear function and the approximated Gaussian prediction is found by fitting the transformed sigma points. Following [49], we use for the unscented transform the parameter values *α* = 0.01, *β* = 2, *κ* = 3−*D* where *D* is the dimension of the state representation inside the UKF.

In the following, we provide an intuitive description of the UKF computations. For the mathematical details, we refer the reader to [49]. The unscented transform is performed twice. First, it is used to approximate the distribution over the decision state in the next time step, as predicted by the generative model from the current estimate based on previous observations, with a Gaussian: $p({\text{z}}_{t}|{\text{X}}_{\Delta t:t-\Delta t})\approx 𝓝({\hat{\text{z}}}_{t},{\hat{\text{P}}}_{t})$. Second, the unscented transform is used to approximate the predicted distribution of the corresponding next sensory observation: $p({\text{x}}_{t}|{\text{X}}_{\Delta t:t-\Delta t})\approx 𝓝({\hat{\text{x}}}_{t},{\hat{\Sigma }}_{t})$. The conceptual idea of Kalman filter algorithms is to compare the predicted distribution with the actual observation and update decision state estimate ${\bar{\text{z}}}_{t}$ proportional to the observed discrepancy while taking the uncertainty over predictions into account. Practically, for the Gaussian approximation used in the UKF we compute a prediction error $\epsilon_{t}={\text{x}}_{t}-{\hat{\text{x}}}_{t}$ between predicted mean ${\hat{\text{x}}}_{t}$ and actual observation **x**_*t*_ and then update the decision state prediction ${\hat{\text{z}}}_{t}$ via a Kalman gain **K**_*t*_:

$${\bar{z}}_{t}={\hat{z}}_{t}+K_{t}\epsilon_{t}.$$ (4)

The Kalman gain represents the relative importance of the prediction errors with respect to the predictions and is computed from the estimated covariance of the predicted observations and the cross-covariance between predicted observations and decision state:

$$K_{t}={\hat{C}}_{t}{\hat{\Sigma }}_{t}^{-1}$$ (5)

where ${\hat{\text{C}}}_{t}$ is the cross-covariance between predicted decision state ${\hat{\text{z}}}_{t}$ and predicted observation ${\hat{\text{x}}}_{t}$ which is strongly affected by dynamics uncertainty *q* (larger *q*, larger cross-covariance) and ${\hat{\Sigma }}_{t}$ is the covariance matrix of the predicted observations which is strongly affected by sensory uncertainty *r* (larger *r*, larger variance). These relations mean that an increase in *q* mostly leads to an increase in gain whereas an increase in *r* leads to a reduction in gain.

**Fig 3:**
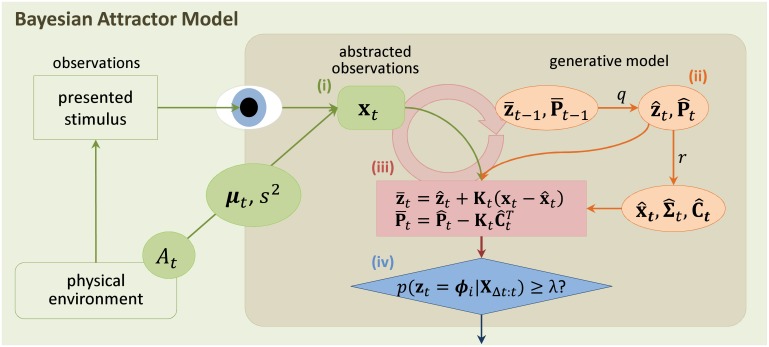
Illustration of the inference scheme used for decision making in the BAttM. In the physical environment a stimulus is presented by the experimenter and observed by the subject. Components inside the shaded rectangle model internal processes of the subject. Sensory processes in the subject’s brain translate the stimulus into an abstract feature representation **x**_*t*_. The input model (i, green) of the BAttM approximates this translation by mapping the stimulus identity (decision alternative *A*_*t*_ at time *t*) to a value **x**_*t*_ drawn from a Gaussian distribution with mean ***μ***_*t*_ and covariance *s* ^2^ **I**. The generative model (ii, orange) states that the decision state **z** is represented by a Gaussian $𝓝({\bar{\text{z}}}_{t-1},{\bar{\text{P}}}_{t-1})$ and evolves according to Hopfield dynamics (Eq 2). The generative model further maps the decision state to different Gaussian densities over observations which mirror those in the input process (Eq 3). Consequently, for the next time step, the generative model predicts the distribution of the decision state, $𝓝({\hat{\text{z}}}_{t},{\hat{\text{P}}}_{t})$, and the distribution of the observation, $𝓝({\hat{\text{x}}}_{t},{\hat{\Sigma }}_{t})$, which critically depend on model parameters *q* and *r*, respectively. The cross-covariance between predicted decision state and predicted observation is denominated ${\hat{\text{C}}}_{t}$. Bayesian inference (iii, red) iteratively compares observations **x**_*t*_ with predictions ${\hat{\text{x}}}_{t}$ and updates the estimate of the decision state (Eq 4) via the Kalman gain **K**_*t*_ which processes the uncertainty defined by ${\hat{\text{C}}}_{t}$ and ${\hat{\text{P}}}_{t}$ (Eq 5). The decision criterion (iv, blue) is defined as a bound *λ* on an explicit measure of confidence (Eq 6).

In addition to affecting the updates of the mean decision state, the Kalman gain is further used to estimate the posterior covariance ${\bar{\text{P}}}_{t}$ of the state variables *z*_*i*,*t*_ which completes the UKF approximation of the posterior distribution over the decision state *p*(**z**_*t*_∣**X**_Δ*t*:*t*_). Fig 3 illustrates the described Kalman filtering scheme.

### Decision criterion

The final component of the Bayesian attractor model is its decision criterion. In decision models based on evidence accumulation the decision criterion *implicitly* sets a particular level of accumulated evidence that needs to be reached before the decision maker commits to a decision. In contrast, we here define the criterion directly on a measure of confidence in the decision. In particular, the model makes a decision for alternative *i* at time *t*, if

$$p(z_{t}=\phi_{i}|X_{\Delta t:t})\geq \lambda$$ (6)

where *p*(**z**_*t*_ = *ϕ*_*i*_∣**X**_Δ*t*:*t*_) is the posterior density over the decision state evaluated at the stable fixed point *ϕ*_*i*_ corresponding to alternative *i*, that is, *p*(**z**_*t*_ = *ϕ*_*i*_∣**X**_Δ*t*:*t*_) is the posterior belief of the decision maker that alternative *i* is the true alternative. Then the threshold *λ* can directly be interpreted as a confidence level. This decision criterion requires that all state variables are at their expected values as given by the stable fixed points *ϕ*_*i*_. Note that this is different from pure attractor models which do not use a bound around the fixed point location, but rather threshold individual state variables *z*_*j*_, see below in results.

**Fig 4:**
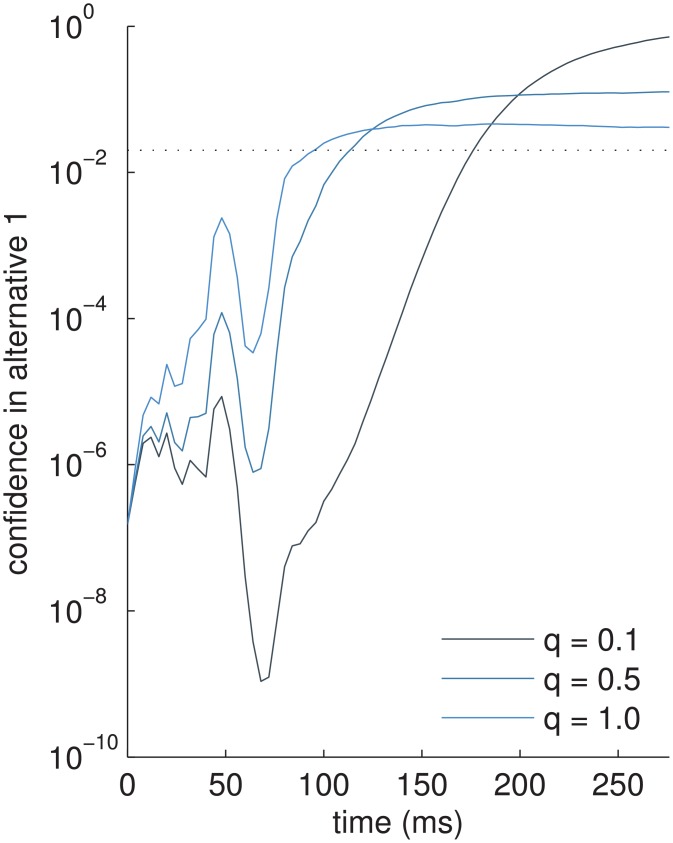
Example trial showing evolution of confidence in alternative 1, *p*(**z**_*t*_ = *ϕ*_1_∣**X**_Δ*t*:*t*_) (notice log-scale and initial, very low values), for different values of dynamics uncertainty *q*. Larger values of *q* mean that only smaller confidence values can be reached even after the decision state **z**_*t*_ eventually settled into the stable fixed point *ϕ*_1_ (compare, e.g., confidence for *q* = 1 and *q* = 0.5 at 200ms, note log-scale). Horizontal dotted line: confidence value used as bound (*λ* = 0.02).

Uncertainty parameters and the confidence bound interact: Larger dynamics uncertainty leads to wider posterior distributions, faster evidence accumulation and smaller density values (Fig 4). For reporting results we therefore fixed the bound to *λ* = 0.02 in all reported experiments which was sufficiently small to be reached for all considered settings of uncertainties. Note that *p*(**z**_*t*_ = *ϕ*_*i*_∣**X**_Δ*t*:*t*_) is not a probability, but a probability density value, that is, it can be larger than 1 and should not be expressed in %. Technically, a probability density value is the slope of the cumulative distribution function of a probability distribution evaluated at a given point in the continuous space over which it is defined.

In the standard, single decision experiments below we report the decision of the first time point for which the decision-criterion (Eq 6) was met. In the re-decision experiment we report the fraction of time in which the criterion was met for the correct alternatives.

## Results

Here we show that the BAttM has ‘inherited’ several key features from the pure attractor model and, in addition, provides for several novel and useful functionalities.

First, we show how the Bayesian attractor model implements the speed-accuracy tradeoff underlying most perceptual decision making experiments. In particular, we show how choice accuracy and mean reaction times can be explained by a combination of input noise level *s* and sensory uncertainty *r* of the decision maker. In other words, we use relative uncertainties to explain specific speed-accuracy tradeoffs. This explanation is a simple consequence of using a probabilistic attractor model in combination with Bayesian inference.

Second, we show that the model can easily explain switches in already made categorical decisions when the sensory input changes. Such re-decisions under uncertainty are often made in our natural dynamic environment but do not seem to be considered by standard experiments and computational models.

Third, we highlight that the BAttM uses a decision state-dependent, top-down modulation of sensory gain such that sensory input affects decisions most, when the decision maker internally predicts the sensory input to be most informative about the decision. Such gain modulation has been hinted at experimentally [39, 40, 53], but has not been considered in the drift-diffusion and attractor models.

Fourth, we show that this formalism enables the explicit computation of confidence in the model. This means that the model not only computes a decision state reflecting the accumulated evidence (as for example in the pure attractor model) but also another dynamic measure, the confidence about making a specific decision. Further, we show that the BAttM can model trial-by-trial variability in confidence judgements as, for example, reported in [41].

Finally, we demonstrate that the BAttM can be used for quantitative analysis of standard perceptual decision making tasks. As an example, we use behavioural data taken from a recent experiment [54] and show that the Bayesian attractor model can fit these data well.

### Speed-accuracy tradeoff in the BAttM

In the BAttM, the speed and accuracy of decisions are primarily controlled by the noise level of the sensory input (*s*), the sensory uncertainty (*r*) and the dynamics uncertainty (*q*). Additionally, the initial state uncertainty *p*_0_ (see Methods) influences the rate of evidence accumulation at the beginning of a trial. First, we demonstrate the effect of the sensory uncertainty *r*, i.e., the decision maker’s internal expectation of how noisy the input is, on decisions.

**Fig 5:**
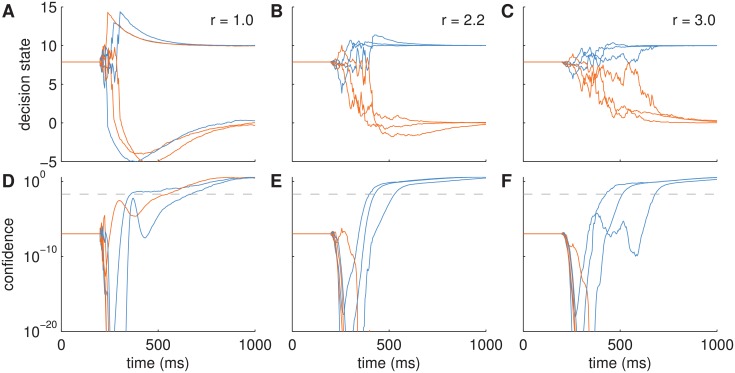
Example trajectories for the Bayesian attractor model on a binary decision task for varying sensory uncertainty *r*. Each of the plots shows three example trials. Note that there are two state variables (blue: alternative 1, orange: alternative 2) for each trial. (A-C) Decision state $\bar{\text{z}}$. (D-F) Confidence (log-scale). Grey, dashed line: threshold used in the model. (A,D) *r* = 1, decisions are inaccurate and shoot over fixed points (located at [10, 0] and [0, 10]). (B,E) *r* = 2.2, decisions are relatively fast and accurate, (C,F) *r* = 3.0, decisions are accurate but can be slow. The same sensory input with noise level (standard deviation) *s* = 4.7 was used in all three cases. Dynamics uncertainty was *q* = 0.1 and initial state uncertainty was *p*_0_ = 5. Note that for clarity we plotted only the mean of the posterior distributions but not the posterior uncertainties (but see below for examples).

Fig 5 shows the dynamics of the decision state over time for three different settings of the decision maker’s sensory uncertainty *r*. After an initial non-decision time of 200ms, the decision variables start accumulating evidence. If the sensory uncertainty is too low, i.e., the decision maker puts too much weight on the noisy input relative to the attractor dynamics (Fig 5A), the decision state overshoots and initially misses the associated fixed point representing a decision. Only after hundreds of milliseconds the decision state relaxes back to a fixed point. This uncertainty setting leads to inaccurate decisions with rather long reaction times. If the sensory uncertainty is too high (Fig 5C), decision making is accurate but relatively slow, because the decision maker expects a much higher noise level than the actual one. When using a suitable sensory uncertainty for the actual noise level of the input (Fig 5B), decision making is fast and accurate as typically observed in subjects.

**Fig 6:**
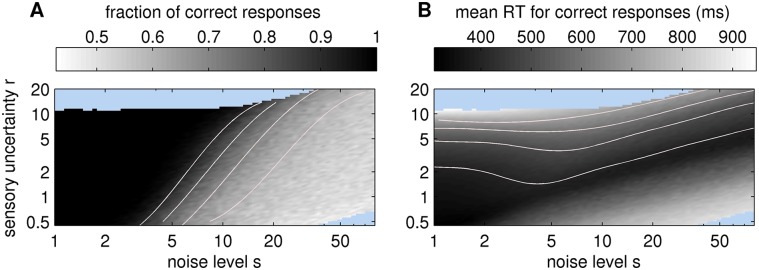
Mapping from sensory uncertainty *r* and noise level *s* to behavioural measures. (A) Log-log plot of the fraction of correct responses, i.e. accuracy. (B) Mean reaction time for correct responses in ms (including a non-decision time of 200ms, see Methods). Light blue areas correspond to parameter settings where more than 50% of trials resulted in time outs (RT >1000ms). Light red lines show approximated contour lines (see Methods of the underlying grey scale map. In A the lines correspond, from right to left, to 0.6, 0.7, 0.8 and 0.9 fraction of correct responses. In B the lines correspond, from bottom to top, to 400, 500, 600 and 700 ms.

To investigate the quantitative dependence of decision state trajectories on both the noise level *s* and the sensory uncertainty *r* we systematically varied these two parameters. We sampled single trial trajectories from each parameter combination while keeping the remaining parameters of the model fixed (*q* = 0.1, *p*_0_ = 5). For more reliable results, we computed the accuracy and mean reaction time over 1,000 single trials for each parameter combination (Fig 6). As expected, the accuracy (Fig 6A) decreases from perfect to chance level as the noise level *s* increases. In general, below *s* < 2, any setting of sensory uncertainty *r* leads to perfect accuracy whereas the mean reaction time (RT) increases with sensory uncertainty *r* (with *r* > 10 RTs can become slower than 1000ms; we excluded these parameter settings from further analysis, see the light blue areas in Fig 6). In contrast, when the noise is large (*s* > 20), the random movement of the dot is too large to recover the stimulus identity reliably, whatever the setting of the sensory uncertainty *r*. For intermediate values of *s*, 3 < *s* < 20, a relatively high accuracy level can be maintained by increasing the sensory uncertainty appropriately; this is reflected by the diagonal gradient between the white and dark grey area in Fig 6A. In Fig 6B there is a narrow valley of fast mean RTs stretching from the lower left to the upper right of the image. Note that the slower RTs below this valley result from trajectories as in Fig 5A. Slower RTs above this valley are due to slow accumulation as seen in Fig 5C. Most importantly, both fast and accurate decisions can be achieved by appropriately adapting the sensory uncertainty *r* to the noise level *s* of the stimulus. The practical use of the results shown in Fig 6 is to fit subject behaviour, i.e., to identify parameter settings which explain the observed accuracy and mean reaction time of a subject.

### Re-decisions

As our environment is dynamic, a specific stimulus may suddenly and unexpectedly change its category. For example, traffic lights turn red and other people may suddenly change their intentions and actions. In these cases one has to make a ‘re-decision’ about the category of the attended stimulus. This is different from the typical ‘single decision’ forced-choice experiments considered in the previous section. These investigate the special case in which the underlying category of a single trial does not change. The corresponding models, like the drift-diffusion model, were designed to model precisely this case and focus on the tradeoff between speed and accuracy of decisions. With re-decisions, another tradeoff, between flexibility and stability in decisions, presents itself. This tradeoff stresses the dilemma of the decision maker to either explain away evidence for an alternative as noise (stability), or rather switch to the alternative decision rapidly (flexibility).

Although one may consider extending the ‘single trial’ models so that re-decisions can be modelled (see Discussion), we found that the BAttM is already an appropriate model of re-decisions. In particular, the sensory uncertainty *r* and dynamics uncertainty *q* are two well-interpretable parameters which control the balance between flexibility and stability. Therefore, the BAttM lends itself naturally as a quantitative analysis method for reaction times and accuracy of re-decisions, as we will demonstrate next.

**Fig 7:**
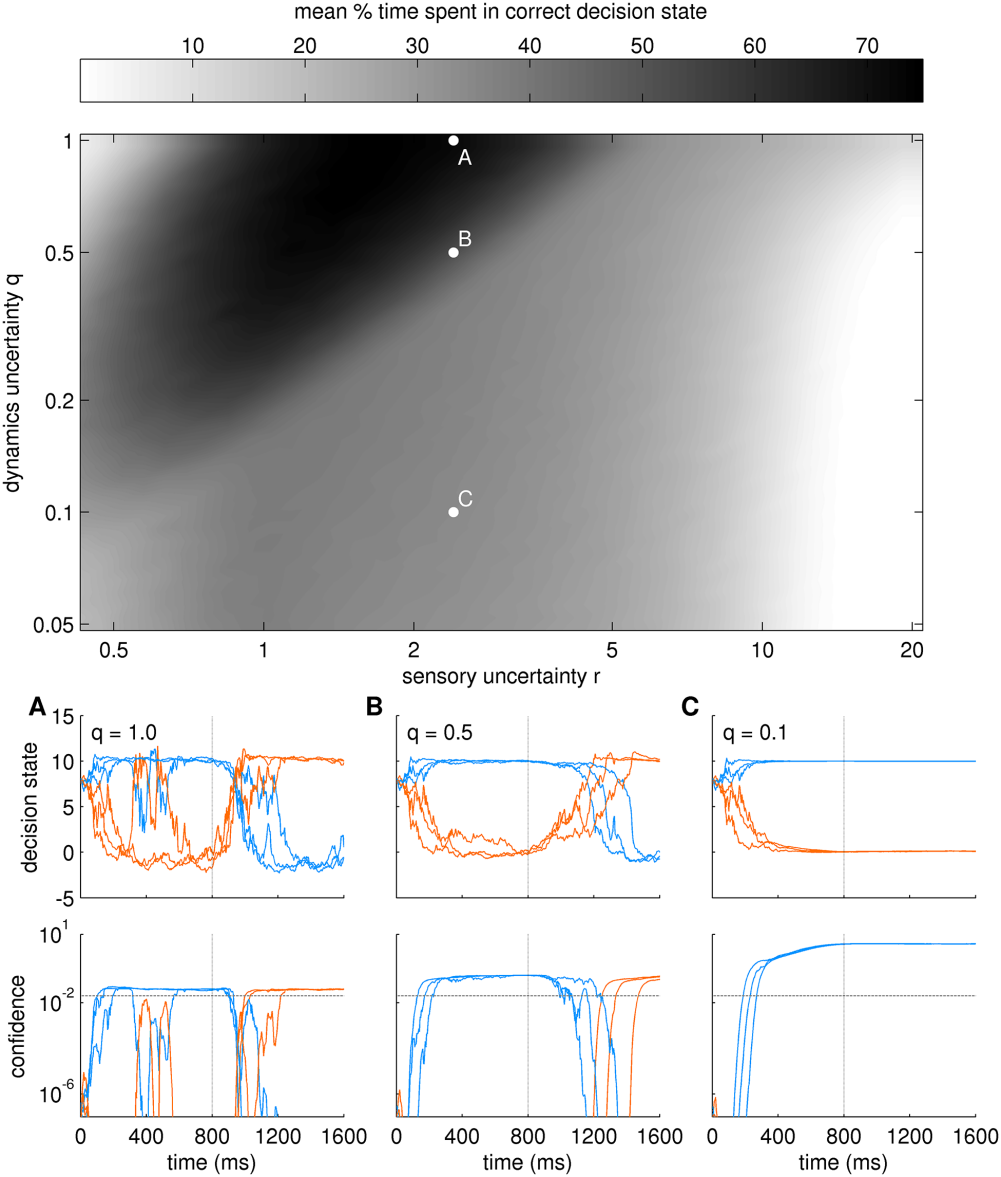
Re-decision behaviour of Bayesian attractor model for switching stimuli. Noisy exemplars of alternative 1 (blue) and subsequently of alternative 2 (orange) were shown with a switch at 800ms (cf. Fig 2). For varying combinations of sensory uncertainty *r* and dynamics uncertainty *q* we plotted the mean (over 1000 trials) percentage of time spent in the correct decision state (grey shading). (A-C) Bottom panels show three example trials for the parameter combinations indicated by the corresponding points in the main panel. Top row: decision state, bottom row: confidence (log-scale) with threshold (grey, dashed line). A: fast, but sometimes fickle re-decisions, B: slower but reliable re-decisions, C: no re-decisions. For point A the mean % time spent in the correct decision is larger, because decision and re-decisions are on average faster. The overall level of confidence reached increases from A to C, as previously shown in Fig 4.

We investigated the re-decision behaviour for a range of parameter settings, see Fig 7. In contrast to the above findings for single decisions, the dynamics uncertainty *q* here plays an important role because it enables the Bayesian attractor dynamics to display different behaviours: When *q* is large, the decision maker will switch readily between fixed points, i.e. decisions. When *q* is small, switching will occur only when sensory input very clearly indicates the alternative. As a proof of principle, we varied the sensory uncertainty *r* and the dynamics uncertainty *q* in logarithmic steps (with fixed noise level *s* = 4), over many (1,000) trials. In each trial, after showing noisy exemplars from one target location (blue alternative) for about 800ms, we switched to the other target (orange alternative) for the same duration (cf. Fig 2).

As a measure for accuracy we report in Fig 7 the mean percentage of time spent in the correct decision state. There are three main regions in the plot: (i) uncertainty settings in the white region lead to extremely slow decisions, (ii) the grey region in which an initial decision (first 800ms) is made but not appropriately updated after a switch and (iii) the black region in which the decision dynamics is sufficiently flexible to make two appropriate decisions. As expected, and in congruence with Fig 6, we find that the sensory uncertainty *r* must be set appropriately (here approximately between 1.5 to 3.0) in relation to the sensory noise level (here *s* = 4.0) to make fast and accurate initial decisions. For further analysis we focus on one of these values (*r* = 2.4), which is consistent with the behavioural data fitting reported below (in our fitting results *r* = 2.4 roughly corresponds to noise level *s* = 4.0 and a coherence of about 25%). We selected three different settings of *q* (0.1, 0.5, 1) as a representative illustration of the results. We display samples of the corresponding trajectories of the decision state in Fig 7A–7C. To compare the impact of the dynamics uncertainty *q*, these samples are based on the same sensory input.

For high dynamics uncertainty *q* = 1.0 (Fig 7A) both the initial decision and the re-decision are made appropriately. However, the decision maker sometimes changes its decision due to sensory noise, i.e., without an underlying switch of stimulus (see Fig 7A at 350ms), exhibiting a high level of flexibility. On average, as re-decisions are made correctly, the performance is relatively large (73%). Although a performance of 73% does not sound very high, it is an open experimental question how human participants would perform in the re-decision experiment. Like the model, a participant will require switching time and may experience transient false beliefs as seen in Fig 7A. In the model, the 73% performance compares well against the two other dynamics uncertainty settings. For example, for a smaller uncertainty (*q* = 0.5, Fig 7B) spurious, noise-induced switches are greatly reduced, but re-decisions are slower. This leads to a reduction in time spent in the correct decision state (53%) in exchange for an increased stability of the decisions. In the grey region (point location and panel C in Fig 7) the dynamics uncertainty is too low (0.1) to make a re-decision based on the sensory input. Only 35% of the time was on average spent in the correct decision state with this setting of *q*, i.e., decisions were detrimentally stable.

In summary, the dynamics uncertainty *q* is a useful parameter for modelling the tradeoff between flexibility and stability of re-decisions. Importantly, similar to the fitting of the experimental data of [54], the mapping of parameters *s*, *r*, and *q* (i.e., noise level, sensory uncertainty and dynamics uncertainty) can be used to quantitatively analyse experimental data in re-decision tasks.

The BAttM suggests an intuitive mechanism of re-decisions: Once an initial decision has been made, the decision state is located in a stable fixed point of the attractor dynamics. As long as sensory observations are consistent with the decision maker’s expectations, the fixed point location is held. When the underlying stimulus changes, however, violated expectations, i.e., large prediction errors (see Fig 1B), force the decision state to move away from the currently occupied fixed point and towards the fixed point representing the identity of the new stimulus, eventually leading to a re-decision. Both sensory uncertainty and dynamics uncertainty control the gain with which prediction errors influence the decision state (cf. Eqs 4 and 5 in models): the sensory uncertainty primarily controls the overall amount of evidence extracted from sensory observations (high uncertainty means low evidence) while the dynamics uncertainty controls how sensory evidence is translated to the decision state (high dynamics uncertainty usually means large effects of sensory evidence on the decision state). Similarly, the gain of the sensory evidence on the decision state is influenced by the decision state itself, implementing state-dependent top-down gain modulation of sensory information. We describe this effect next.

### Top-down gain modulation

There is growing evidence that higher level cognitive processes modulate neural responses already in early sensory areas [36–38, 55–58]. More specifically, recent findings [39, 40, 53] indicate that neural activity in early sensory areas is modulated by the final choice of subjects in simple perceptual decision tasks. These findings suggest that top-down feedback influences sensory processing already on the temporal scale of single decisions, i.e., within a trial of a perceptual decision making task. Pure attractor and drift-diffusion models, however, do not account for top-down feedback that modulates the extraction of evidence on the sensory level. In this section, we show that the BAttM offers such a top-down computational mechanism that leads to a stabilisation of the fixed points of the attractor dynamics and, consequently, allows the decision maker to make confidence-informed decisions.

This mechanism can be best understood by comparing the within-trial dynamics of the decision state for both pure attractor models (Eq 1) and the BAttM. Bayesian inference in the BAttM implements a predictive coding scheme (Eq 4) in which state predictions ${\hat{\text{z}}}_{t}$ are updated with information from prediction errors ***ϵ***_*t*_ dependent on a Kalman gain matrix **K**_*t*_ (Eq 5) which embodies uncertainty and the relation between observations **x** and decision variables **z**. To compare the pure attractor model with the BAttM we first note that both models have the same form: After approximating the mean state prediction ${\hat{\text{z}}}_{t}$ with the (expected) attractor dynamics of the generative model,

$${\hat{z}}_{t}\approx {\bar{z}}_{t-\Delta t}+\Delta tf\left({\bar{z}}_{t-\Delta t}\right),$$ (7)

we can plug this approximation into Eq (4). The resulting Bayesian inference formalism replicates the form of the attractor model in Eq (1):

$${\bar{z}}_{t}-{\bar{z}}_{t-\Delta t}\approx \Delta tf\left({\bar{z}}_{t-\Delta t}\right)+K_{t}\epsilon_{t}.$$ (8)

The critical difference of the BAttM formalism of Eq (8) to the pure attractor model in Eq (1) is that the BAttM prescribes an input consisting of a prediction error scaled by the gain. In particular, the input to the Bayesian attractor model depends on the last state ${\bar{\text{z}}}_{t-\Delta t}$ both through the gain matrix **K**_*t*_ and the mean prediction ${\hat{\text{x}}}_{t}$ (see Models). This means that sensory observations pass through two processing steps which are applied in each time step: (i) Computation of prediction error using the top-down prediction, and (ii) modulation of the prediction error by the gain which also translates the sensory information (prediction errors) into the decision space (through linear transformation by the gain matrix **K**_*t*_).

In this model, the effect of the gain is driven by two opposing components: In general, when predictions are more certain, the gain is increased. This effect is primarily mediated by the uncertainty *r* at the sensory level. Importantly, the gain is also driven by the cross-covariance of the predicted decision state ${\hat{\text{z}}}_{t}$ and predicted sensory observations ${\hat{\text{x}}}_{t}$ (Eq 5). The cross-covariance describes the information about changes in the decision state that can explain variation in sensory observations. It defines how prediction errors in sensory observations induce necessary changes in the decision state. This effect is largest in the space between fixed points of the attractor dynamics, because here a change in the decision state almost linearly maps to a change in sensory predictions. In contrast, the effect is relatively small close to the fixed points (see Methods for details). As uncertainty in the decision state increases, it becomes more likely that the underlying distribution covers more of the space between fixed points, thereby increasing cross-covariance. Consequently and opposite to uncertainty at the sensory level, higher uncertainty at the decision level typically leads to larger top-down gain.

**Fig 8:**
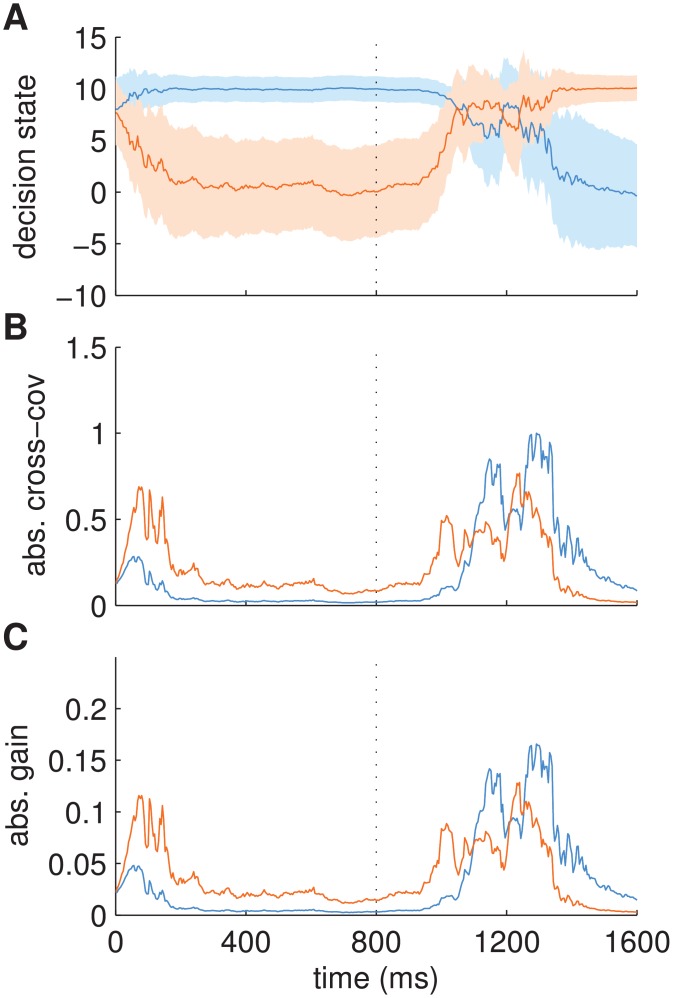
Example of a decision making trial with evolution of cross-covariance and gain for parameters of point B in Fig 7. Noisy exemplars of alternative 1 (blue) and subsequently of alternative 2 (orange) were shown with a switch at 800ms (cf. Fig 2). (A) Inferred decision state with mean state variables (lines) and two times their standard deviation (shading) indicating posterior uncertainty over decision state. State variable associated with alternative 1 shown in blue and associated with alternative 2 shown in orange. (B) Absolute cross-covariances between predicted observations and predicted decision state over time. Colours indicate cross-covariances associated with corresponding state variables as in A. Cross-covariances are large during their transition between fixed points. Once a fixed point is reached (i.e. a decision has been made) cross-covariances drop quickly. (C) Absolute gain values (elements of **K**_*t*_) over time. Colouring as in B. Gain values are scaled cross-covariances, i.e., within-trial changes in gain are mostly driven by changes in cross-covariances.

Fig 8 demonstrates this within-trial gain modulation mediated by cross-covariance, for the empirically inferred parameters of point B of Fig 7 (*s* = 4, *r* = 2.4, *q* = 0.5). Fig 8A shows the inferred decision state as a function of time. After the switch of the stimulus, between 800 and 1,500ms, the decision state moved between fixed points of the attractor dynamics. As can be seen in Fig 8B, the predicted cross-covariances between decision state and sensory observations were large during this time period and became small again once the dynamics settled into a fixed point after 1,500ms, i.e., when a decision had been made. Similar dynamics can be seen for the initial decision around 0 to 200ms. Fig 8C plots the elements of the gain matrix **K**_*t*_ over time. The trajectories follow those of the cross-covariance closely demonstrating that within-trial changes in gain were driven nearly exclusively by changes in the cross-covariance. Although the uncertainty over the decision state also varied within the trial (Fig 8A, shading), the effect on the uncertainty of predicted observations was small in comparison to the effect exerted by the sensory uncertainty *r*, which remained constant throughout the trial.

In summary, the within-trial, state-dependent modulation of gain is a useful mechanism when making decisions: It stabilises the representation of the stimulus category (low gain close to fixed points, see below), but also implements fast accumulation of evidence, when needed (high gain between fixed points).

### Confidence-based decision criterion

A graded feeling of confidence appears to be a fundamental aspect of human decision making. Corresponding confidence judgements can inform about underlying decision processes [42, 43]. Through the probabilistic formulation, the BAttM directly provides a continuous measure of confidence that may be compared to experimentally measured confidence judgements. In the following we describe how confidence is computed in the BAttM, explain its use within the decision criterion and demonstrate that it conforms to experimental findings about confidence judgements [41, 42].

The substantial and sudden decrease of gain close to a fixed point (e.g., Fig 8C, at 1,400ms) contributes to an important feature of the BAttM: The location of fixed points is the same for different stimulus strengths. As we will show in this section, stable fixed point locations are the basis for defining a decision criterion directly on an explicit measure of confidence.

**Fig 9:**
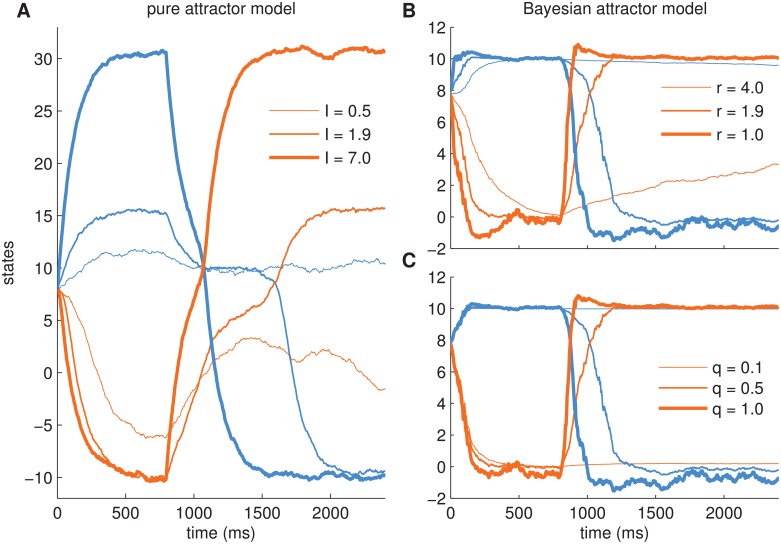
Evolution of decision state for pure attractor model (left) and Bayesian attractor model (right) for different input strengths or different uncertainty parameters, respectively. There are two alternatives indicated by blue (alternative 1) and orange (alternative 2). Thinner lines indicate smaller stimulus strength. For the first 800ms, input reflecting alternative 1 was shown, with a switch to input caused by alternative 2 at 800ms. (A) In the pure attractor model speed and accuracy of initial and re-decisions is controlled by the input which we set to **I**_*t*_ = [Δ*tI*+*v*_*t*_,0], if alternative 1 is correct, and **I**_*t*_ = [0,Δ*tI*+*v*_*t*_], if alternative 2 is correct (*v*_*t*_ ∼ 𝓝(0,0.2^2^)). We varied the value of *I* as indicated in the plot legend. If *I* is large, i.e., the task is easy, initial decisions and switches are fast (thick lines). The position of the fixed point, to which the dynamics converges, depends strongly on *I*. (B, C): In the Bayesian attractor model timing and accuracy of initial decisions and re-decisions depend on the uncertainties in the model, but, critically, the location of the fixed points of the dynamics remain the same for different uncertainties. B and C share the same observations with noise level *s* = 1. In B: *q* = 0.5. In C: *r* = 1.9.

Pure attractor models do not have stable fixed points: Because noisy evidence directly feeds onto the decision variable (see Eq 1 and Fig 1A), the location of fixed points depends on the magnitude of the evidence, i.e., stimulus strength. We show this effect in Fig 9A, see also [59]. Therefore, in pure attractor models, as long as stimulus strength is assumed to be unknown, one cannot tell how close the current decision state is to a fixed point, that is, fixed points have no particular meaning in pure attractor models except that the dynamics will eventually converge to them.

In contrast, in the BAttM the speed of evidence accumulation, as caused by a particular, underlying stimulus strength, can vary without affecting fixed point locations (Fig 9B and 9C). This is because the BAttM implicitly represents stimulus strength in its uncertainty parameters *r* and *q* such that *expected* stimulus strength is automatically taken into account during evidence computation from the stimulus. As a consequence of stable fixed point locations, a deviation of the decision state from a fixed point can be readily interpreted as violation of the expectations about the stimulus associated with that fixed point, irrespective of stimulus strength. In general, the more such expectations are violated, the less confident the decision maker should be about choosing one of the alternatives. We implemented this mechanism in the BattM by deriving the confidence in a decision alternative directly from the probabilistic model and using a threshold on it as decision criterion (see Models, Eq 6).

**Fig 10:**
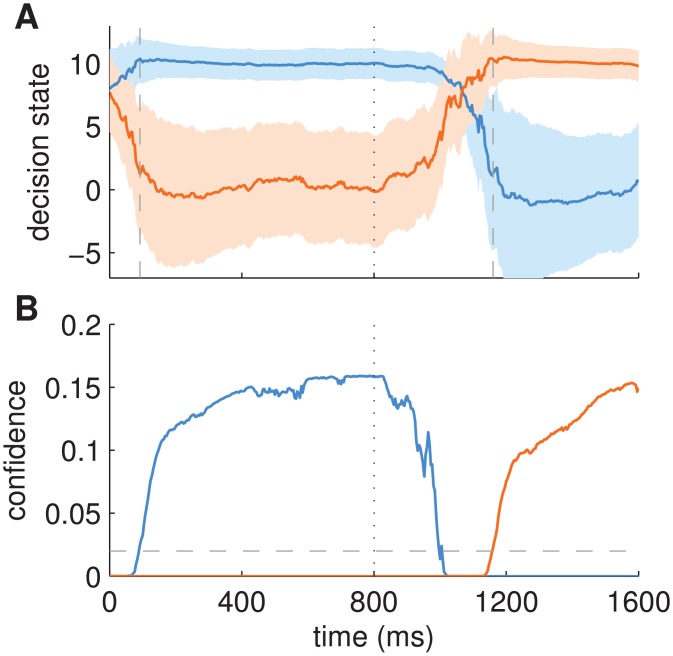
Example evolution of the posterior density of the decision state and the associated confidence values for one trial with a switch of stimulus at 800ms (vertical, dotted line). (A) Posterior density of the decision state with mean (coloured lines) and two times standard deviation (shading) of decision state variables as in Fig 8A. Grey, dashed lines in A show the decision times for the initial decision (92ms) and the re-decision after the switch (1160ms). (B) Solid lines indicate confidence values for both alternatives, i.e., the posterior probability density values that the decision state is in one of the stable fixed points of the attractor dynamics. The decision threshold is indicated as grey, dashed line. The parameters of the model were those of Fig 7B (*r* = 2.4, *s* = 4, *q* = 0.5).

In Fig 10 we illustrate how confidence values relate to the posterior density of the decision state (Fig 10A), and how confidence-based decisions are made (Fig 10B). Intuitively, the confidence for a specific alternative measures the distance of the current decision state (blue and orange lines in Fig 10A) from the stable fixed point of that alternative (at [0, 10] or [10, 0]) scaled by the posterior uncertainty of the decision state. Consequently, the confidence for all alternatives can be tracked across time (cf. blue and orange lines in Fig 10B). Strikingly, the confidence dynamics are different from the decision variable dynamics: While the decision state gradually moves towards a fixed point, thus reflecting the relatively slow gradual accumulation of evidence (e.g., time period 800 to ∼ 1100ms), the confidence rises abruptly as soon as the posterior density of the decision state starts concentrating around a fixed point (e.g., from ∼ 1100ms onwards).

**Fig 11:**
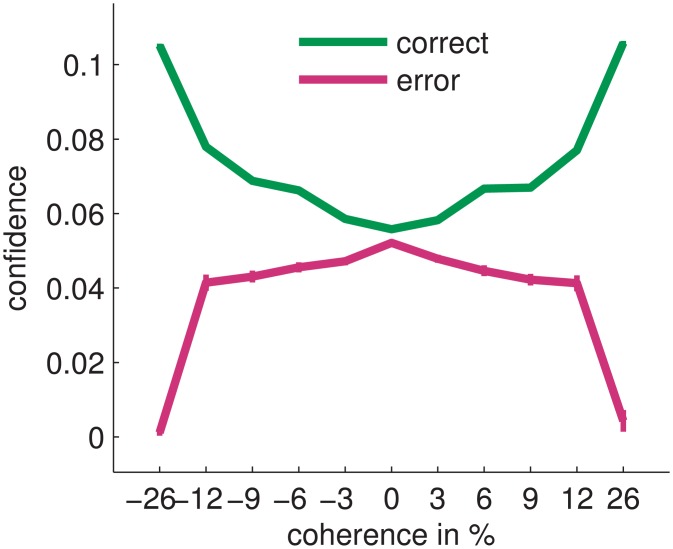
Confidence in relation to stimulus strength as predicted by the BAttM for the experiment of [54]. These confidence values result from continuing accumulation of evidence for 100ms after the internal threshold was crossed but before a corresponding motor response was completed (cf. [35]). Negative coherences: left motion stimulus, positive coherences: right motion stimulus. For each coherence level we simulated 2,500 trials (5,000 for 0% coherence) using the BAttM. Shown are mean confidence values and their standard errors. Parameters were those listed in Table 2 with *q* = 0.5.

How does the confidence-based decision making formalism compare with experimental findings? Early behavioural work with humans [42], indirect confidence judgements by rats [41] and general theoretical considerations [42, 43] suggest that confidence in correct choices increases with stimulus strength whereas confidence in erroneous choices decreases with stimulus strength. At first glance, this seems at odds with a confidence-based decision criterion, as used by the BAttM, where the decision is made exactly when the confidence is at a specific level, independent of stimulus strength (Fig 10B). This apparent contradiction can be resolved by noting that subjects, in the typical experimental setup, keep observing the stimulus for a short time after reaching the threshold, because of the delay between an internal decision and the production of the corresponding motor output, such as a button press. In standard models, this time period is usually considered to be part of the total non-decision time. Importantly, the same mechanism of continued accumulation of evidence in this time period is thought to contribute to ‘changes of mind’ observed in a reaching task [35] where subjects revise their internal categorization before being able to fully execute the reaching movement. We implemented this mechanism in the BAttM by continuing the accumulation of evidence after crossing the confidence threshold for about half of the estimated non-decision time of 200ms, i.e., for 100ms. Critically, during this continued accumulation period, the confidence values evolve further and replicate the reported experimental results that show a dependence of confidence on stimulus strength and correctness of decision (Fig 11).

### Fitting of a reaction time experiment

**Fig 12:**
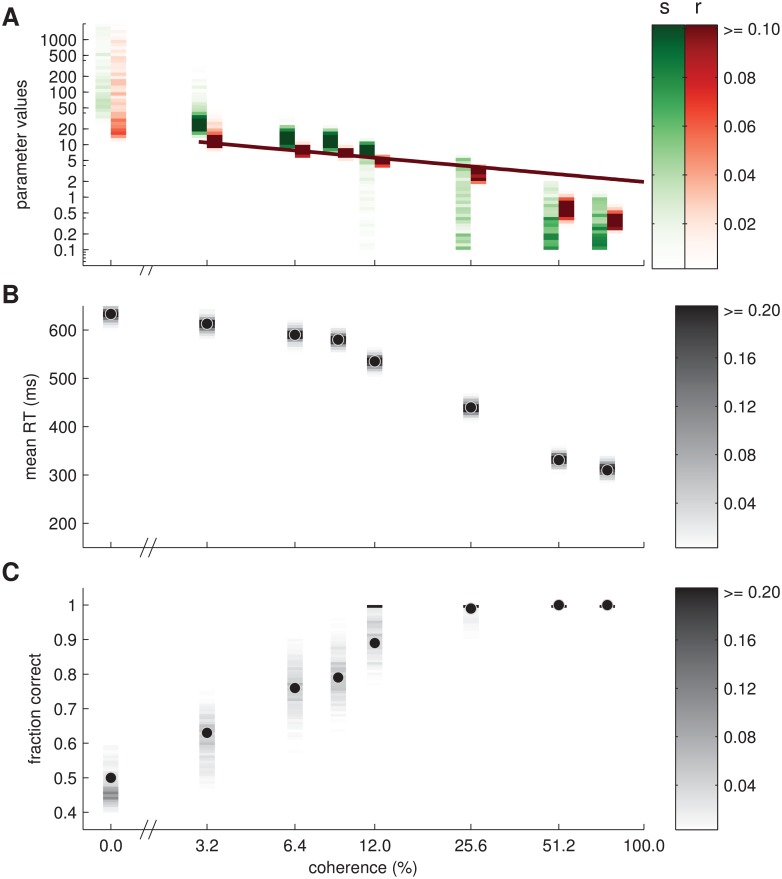
Model fit to experimental data presented in [54]. Eight different coherence levels ranged from 0% to 75%. (A) Model parameters (red: sensory uncertainty *r*, green: noise level *s*) inferred from the behavioural data. For each coherence and parameter we show an approximate posterior distribution estimated from 501 posterior samples (see Methods) where darker colours correspond to larger probability as indicated by the colour bars on the right. Both abscissa and ordinate are in log-scale. Red line: linear fit between sensory variance *r* ^2^ and coherence that also exposes a linear relation between drift and coherence in the drift diffusion model. (B) Fit of mean RT of all responses. Black dots with light grey outline: behavioural data [54]. Greyscale rectangles: estimated posterior distribution over mean reaction time. (C) Fit of accuracy (fraction of correct responses). Format as in B. Black, horizontal bars for coherences greater than 9% indicate probabilities larger than 0.2 for an accuracy of 1. This means that for high coherences parameter values as indicated in A predicted an accuracy of 1.

To establish the validity of the proposed model and show that the model can be used to analyse data of decision making tasks, we fit behavioural macaque monkey data on the RDM two-alternative forced choice task presented in [54]. These authors used a drift-diffusion model to fit the average responses based on 15,937 trials. Stimuli were presented at eight different coherence levels ranging from 0% to 75%. We extracted the averages of the behavioural data from Figure 1 d,f in [54] and re-plotted the data in Fig 12B and 12C (black dots). We obtained the model fit by stochastically minimising an objective function which quantified the discrepancy between values sampled from the model and the behavioural data (cf. Methods). The sampled RTs contained a non-decision time which was reported in [54] (see Methods for details). We plot the fits of mean reaction time and accuracy in Fig 12B and 12C. In Fig 12A, we show the fitted model parameters, noise level *s* and sensory uncertainty *r*, see also Table 2.

**Table 2:** Fitted parameter values (best fitting sample for each coherence).

**coherhence (%)**	**0**	**3.2**	**6.4**	**9**	**12**	**25.6**	**51.2**	**75**
**sensory uncertainty *r***	18	11.2	7.4	6.7	4.8	2.3	0.55	0.30
**noise level *s***	56.9	23.7	13.6	11.9	8.5	3.8	0.16	0.14

These results demonstrate that the model can fit the mean RTs and accuracy for different coherence levels by varying the sensory noise and the internal uncertainty of the decision maker. As can be seen in Fig 12A and Table 2, we found, as expected, that both the sensory uncertainty and the noise level decrease as a function of coherence. The estimated posterior parameter variances indicate that parameters of the model can be estimated reliably for intermediate accuracies. When accuracy reaches its ceiling at 100% for coherences greater than 25% many different noise levels *s* can lead to equivalent predictions, simply because noise is not needed anymore to explain erroneous choices and can be set arbitrarily small.

It has previously been found that the drift in a drift diffusion model scales linearly with coherence (e.g., [54]). We found an equivalent relation between the sensory uncertainty *r* and coherence (Fig 12A, red line). In particular, it has been shown for a simple probabilistic model ([23], Eq 22) that sensory uncertainty *r* relates to drift *v* in the drift diffusion model as *r* ^2^ = 2/(*v*Δ*t* ^2^). If *v* = *Kc* as in [54], *r* can be written as *r* ^2^ = *K*′/*c*. We applied this relation to the BAttM and fitted *K*′ to the values of *r* reported in Table 2 (see Methods for details). The result captures the previously reported relation between coherence and sensory uncertainty well for most coherences (red line in Fig 12A) and only deviates from the fitted parameter values for coherences greater than 25%; see Discussion for a potential, interesting reason.

In all work presented here we fixed the confidence threshold *λ* to a constant value. This was necessary, because *λ* and sensory uncertainty *r* have very similar effects on mean RT and, thus, are interchangeable in many conditions (cf. [23]). To verify this relationship we repeated fitting of the data used here, but fixed *r* = *s* and allowed *λ* to vary. With this parameterisation, we could fit behaviour for high and intermediate coherences equally well, but observed a drop in quality of fit for low coherences (0% and 3.2%, results not shown).

## Discussion

We have embedded an attractor model into a Bayesian framework, resulting in a novel Bayesian attractor model (BAttM) for perceptual decision making. The model can be used as an analysis tool to fit choices and response times of subjects in standard perceptual decision making tasks (Table 2, Fig 12). It also extends to re-decision tasks where the participant has to detect stimulus changes and make another decision (Fig 7). In addition, the model predicts state-dependent, within-trial gain modulation of sensory processing by top-down feedback of the decision state (Eq 8, Fig 8). This top-down gain modulation enables an explicit measure of confidence in decisions (Fig 10) that reproduces recent experimental findings about confidence judgements in perceptual decision tasks (Fig 11).

### Re-decisions

In typical perceptual decision making experiments, e.g. [54], the response of the participant automatically ends a trial and the stimulus disappears. In natural conditions, however, an object typically does not disappear after the brain has made its categorisation and the object should be represented as long as it is behaviourally relevant. In addition, the brain has to be able to rapidly update a decision in response to a change in the environment, for example, when a green traffic light turns red. These decisions, which we called re-decisions, are currently rather not considered by perceptual decision making models. In particular, drift-diffusion and similar probabilistic models of perceptual decisions are not good models for behaviour in response to stimuli that switch occasionally. This is simply because the amount of accumulated evidence for a decision depends on the time the stimulus supporting the decision is observed: To switch to the alternative decision, this accumulated evidence must be overcome by an equal amount of evidence in favour of the alternative. This means that the reaction time in response to a switch would depend on how long the previous stimulus was shown. If the previous stimulus was present for several seconds, standard drift-diffusion and related models predict that the reaction time for a switch would be several seconds as well. This would clearly depart from the expected decision behaviour of participants with typical reaction times of several hundred milliseconds.

Pure attractor models, on the other hand, provide a basis for successful re-decisions: Once the decision state is in a fixed point no additional evidence is accumulated. Consequently, only a fixed amount of evidence for the alternative category is required to reverse an initial decision by moving the decision state into a different attractor [26]. The BAttM enhances this property through its embedding in a probabilistic framework: It provides a single, interpretable parameter, the dynamics uncertainty *q* (cf. Table 1), that controls the timing of re-decisions independently of the timing of initial decisions and, thus, implements a tradeoff between flexible and stable decisions (Figs 7, 9C).

Note that the drift diffusion model could be extended to allow for re-decisions that do not depend on the duration of the previous stimulus. In a neural model of a drift diffusion process this could be achieved by using neurons with a maximal firing rate. In mathematical formulations based on a stochastic differential equation [6, 20], such a maximal firing rate mechanism translates to a condition which would increasingly limit the size of state changes as the maximum state value is approached. To the best of our knowledge, such a mechanism has not been described yet and would reproduce a key feature of attractor models where state changes decrease as a fixed point is approached.

So-called changes of mind [31, 35] differ from re-decisions. In [35] a change of mind occurred very quickly to correct an initial decision, that is, without a change of stimulus subjects changed their decision, presumably, in response to stimulus information that was processed just after the initial decision had been made. In contrast, re-decisions can also occur long after a decision that was made with high confidence. Specifically, the model of changes of mind described in [35] extended a standard drift-diffusion model with an additional bound which only comes into effect after one of the initial bounds has been crossed, that is, after an initial decision has been made. This second bound is only defined for the initially unchosen alternative. Other than in the standard drift-diffusion model, accumulation of evidence continues after the decision. If the second bound is reached within a given deadline, a change of mind is executed. There are two properties of this model which prevent modelling re-decisions in response to a change in stimulus: 1) the deadline and 2) (as described more generally for drift diffusion models above) the dependence of re-decision times on the time of the underlying stimulus switch. The deadline in the change-of-mind model was designed to capture motor costs that prevent a change-of-mind too close to the end of the trial. The deadline, therefore, could simply be dropped in a re-decision experiment. However, the more general drawback of drift diffusion models, i.e., the dependency of re-decisions on the duration of the previous stimulus, would have to be fixed more elaborately (see previous paragraph).

To investigate re-decisions in experiments, standard perceptual decision making paradigms need to be adapted. Especially, single trials need to be prolonged in order to present changing stimuli to the participants and allow them to react to these changes.

### Benefits of a probabilistic formulation

As stated above, although there may be differences in detail, pure attractor models can, in principle, explain re-decisions as well. One question is what the BAttM can offer beyond what pure attractor models can do. An important advantage of a probabilistic formulation is that it allows to define confidence measures, as discussed further below. Another crucial advantage is that the BAttM explicitly models how evidence for a decision is extracted from the concrete features of a given stimulus. This means that the BAttM can in principle predict reaction times and choices of the subject given the stimulus features of the actual stimulus shown to the subject in each single trial. Although this may appear as a technical detail, we believe this input model (see Fig 3) is a vital model component. For example, pure attractor models require that the modeller provides the evidence input. This ‘manual’ specification of the evidence input is not necessarily an advantage because the exact shape of the input is a key to explain the data. This would make the manual input specification an important but rather ill-constrained component of the model as there is no measure of the degrees of freedom spent on the input specification. In contrast, the BAttM explicitly constrains evidence computation via the Bayesian update equations. As a result, stimulus features shown to the subject enter the behavioural analysis in a highly constrained fashion. This formally described evidence computation also defines the top-down modulation predicted by the BAttM, as discussed next.

### Uncertainty and top-down modulation

In the BAttM, there are two different ways how top-down gain modulation of sensory processing emerges. The first depends on the sensory uncertainty *r*, which we implicitly assume here is a between-trial effect because most experiments keep the amplitude of the sensory noise constant over a trial, but see ‘Adapting stimulus expectations’ below for a discussion of this assumption. The second gain effect varies due to the dynamics of the internal decision state, which is a within-trial modulation.

The between-trial gain modulation offers a novel understanding of variations in reaction times caused by varying stimulus noise level. In explanations of perceptual decision making it is generally assumed that stronger stimuli, i.e., with higher signal-to-noise ratio, translate into larger pieces of evidence which lead to faster accumulation [1]. The BAttM makes this translation explicit and models higher stimulus strength by less observation noise *s* and correspondingly less sensory uncertainty *r* (Table 2, Fig 12). A key prediction of the BAttM is that different speeds of evidence accumulation, e.g., across task difficulty levels, are caused by different amounts of top-down gain modulation: the lower the sensory uncertainty, the higher the gain of sensory input (Eq 5). Such a top-down mechanism has been described in general by proponents of the Bayesian brain hypothesis [45, 46, 60], the free energy principle [61] and predictive coding [62]. In particular, it has been suggested that internal uncertainty is tightly linked to neuronal modulator mechanisms [63–65] that implement attentional, top-down modulation of sensory areas [36–38, 55–58].

In addition to these between-trial effects, experimental findings prompted the suggestion that sensory gain may be modulated within-trial by the state of an ongoing decision [39, 40, 53]. Drift-diffusion and pure attractor models do not account for such top-down modulation of gain, because there is no top-down connection from decision state to sensory input in these models. In the BAttM, however, this connection is provided by the state-dependent Kalman gain, see Eqs (8, 5). In particular, the BAttM predicts that sensory gain is large when transitioning between decision alternatives and small when the decision is imminent or has been made (Fig 8). This modulation is driven by the cross-covariance between predicted observations and decision states (Fig 8). Intuitively, this cross-covariance measures what changes can be expected on the observation level due to a change of the decision state, or, inversely, what changes in the decision state are likely to explain changes on the observation level. Therefore, the described formalism underlying within-trial gain modulation differs from the between-trial modulation which is purely based on changes in sensory uncertainty.

Previous experiments [39, 53] showed only coarse-grained evidence for decision-dependent modulation of activity in sensory areas, or are currently difficult to translate into our formalisation due to the type of measurement [40]. Therefore, further research is needed to test the hypothesis of specific temporal structure of gain modulation as predicted by the BAttM. Note that the BAttM was not designed by us to employ such a state-dependent top-down modulatory mechanism; rather, this property emerges from the Bayesian formulation in which decision states explicitly connect to particular sensory observations. Furthermore, the gain modulation in the BAttM has two functional benefits: First, it leads to a common, stable representation of the decision across task difficulties while still allowing decisions to be made with varying accuracy and timing. This is not the case for pure attractor models (Fig 9) but is useful for a neuronal implementation because the next higher level can more easily read out a stable representation. Second, within-trial gain modulation facilitates rapid updating of decisions in response to a changed stimulus, because it quickly destabilises a made decision when sufficient evidence to the contrary is available. Consequently, the increased gain speeds up the transition to an alternative decision. Note that the initial movement out of a fixed point that represents a previously made decision is mediated by prediction errors (Eq 8) which tend to be large when the decision deviates from the real stimulus and small otherwise.

Although there are some reports of potential within-trial top-down gain modulation [39, 40, 53], the formalism implemented by the BAttM is, at the current time point, a purely theoretical prediction which may be tested in future experimental work. Diffusion models often successfully explain decision behaviour without using top-down feedback mechanisms. Therefore, it may appear that the brain does not use top-down feedback when making simple perceptual decisions. However, a simple experiment testing the existence of top-down modulation may proceed as follows: Participants would be cued about the upcoming stimulus strength only in some trials but not in others. If the predictive cue had an effect on decisions, the BAttM would predict that this was partially due to between-trial top-down modulation through updated expectations of the participants. It is harder to test the existence of within-trial top-down modulation that discriminates the BAttM from pure attractor and diffusion models. Novel tasks may be required to elicit measurable effects of such within-trial top-down modulation. For example, the BAttM predicts that top-down modulation varies strongly in experiments with longer trials including re-decisions. In addition, the BAttM could be used to test this particular question by removing within-trial top-down gain modulation in the model and comparing choices predicted from this reduced model with those predicted from the full BAttM.

### Confidence-based decisions

“It has been definitely shown that the recognition process is attended by varying degrees of confidence; that the correctness of recognition tends to vary directly with the degree of confidence, and that our belief-attitudes appear with varying degrees of strength, or varying degrees of confidence, assurance, or certainty.” [66] Since 1926 this account has been consolidated and given a theoretical basis [42]. More recently, behavioural paradigms were developed in which confidence could be measured from non-verbal responses [41, 67]. These developments have been accompanied by extensions of drift-diffusion and attractor models that explain measured confidence ratings: For drift-diffusion models explicit confidence values can be computed as function of the decision variable and time [67] under the assumption that subjects’ confidence equals their true probability of making an error, but see [68]. Alternatively, the decision variable itself can be related to subjective confidence in the drift-diffusion model [23]. In pure attractor models, the decision state has been related to confidence judgements only indirectly: The increasing magnitudes of the decision state at the fixed point locations for increasing stimulus strengths (cf. Fig 9A) have been interpreted as increasing confidence in the decision [59]. This account assumes that the decision state continues to evolve towards the fixed points of the dynamics after the decision threshold has been reached.

Other than both drift-diffusion and pure attractor models, the BAttM computes an explicit (i.e., in addition to the decision state) and ongoing measure of confidence based on subjective uncertainties of the decision maker (see Fig 10 and Fig 4). This enables us to model confidence-based decisions using a threshold on the ongoing confidence (Fig 10B) which, in the BAttM, is defined as the posterior density that the decision state is in a stable fixed point of the generative model (cf. Eq 6 in Methods). This posterior density can be interpreted as the decision maker’s internal belief that a category is the true category of the stimulus and can be easily computed from the estimated posterior over the decision state for an arbitrary number of alternatives. Note that the threshold on confidence may be implemented by a simple threshold on firing rates of neurons that represent the corresponding posterior density. As a density, however, it cannot be expressed in percent and, therefore, lacks an intuitive connection to typical measures of confidence in behavioural experiments. This connection may instead be provided by alternative measures of confidence that can also be derived from the posterior distribution over the decision state. For example, one can compute, as a measure of confidence, the probability that any one of the decision state variables exceeds all other state variables. This probability can be expressed in percent. It is possible that subjects compute such a measure when asked to explicitly report confidence after the decision, but it is an open experimental question how to identify forms of confidence judgements actually used by the brain.

As the BAttM uses a threshold on the confidence to make a decision, the confidence at decision time is always equal to the threshold. This fact appears to contradict key experimental findings showing a dependence of confidence judgements on decision outcome and stimulus strength [42, 43]. Yet, this apparent mismatch can be resolved (Fig 11) simply by continuing accumulation of evidence during part of the non-decision time period. This continued accumulation is motivated by a corresponding assumption in [59] and by recent experimental findings regarding changes-of-mind in decision making [35]. It has also been shown that a wide range of findings about confidence ratings can be replicated under the assumption that evidence accumulation continues until the confidence rating [69]. In further congruence, potential neural correlates of continued processing of the stimulus after reaching a threshold were reported in [70].

Furthermore, the BAttM predicts direct, intuitive relations between the internal uncertainties of a decision maker and the absolute level of confidence that can be reached: Larger uncertainties lead to smaller confidence (e.g., see Fig 4). As these uncertainties simultaneously control choices, response times and re-decision times, we propose to validate the consistency of these predicted relations in future experiments.

### Interpretation of the fit to [54]

We fitted the BAttM to average behaviour reported in [54] and found that the BAttM explains decision making behaviour well (Fig 12B and 12C) even though we assumed a simplified representation of the stimulus (cf. section input). This was expected, because 1) a similar, abstract stimulus representation was sufficient to fit behavioural data (of humans) before [23] and 2) [54] originally used a similar computational representation to fit a drift-diffusion model to the data considered here.

For the BAttM, estimates of the reliability of parameter fits indicate that fitted parameter values are highly reliable for experimental conditions in which subjects exhibit intermediate accuracy in response to coherences from 3.2% to 12% (Fig 12A). In these conditions our fits suggest that the noise level *s* exceeded sensory uncertainty *r* in the subjects which would mean that the subjects’ generative model of the stimulus underestimated the amount of noise in the stimulus. In contrast, an optimal Bayesian decision maker should have a generative model in which, ideally, *r* would equal *s*. It has been proposed that variability in subjects’ responses may be due to suboptimal inference [71], that is, inference based on suboptimal, or wrong assumptions about the underlying statistical structure of the inference problem. Our observation that *s* exceeds *r* suggests that subjects indeed perform suboptimal inference in the corresponding choice task. This finding, however, only holds under the assumption that the confidence threshold is set to a constant, low value (*λ* = 0.02), because *r* and *λ* have very similar effects on accuracy and mean RT. Indeed, we also found that behaviour in most conditions could be fit equally well, when *r* was constrained to be equal to *s*, but *λ* was allowed to vary freely. Although the drop in quality of fit for coherences 0% and 3.2% (cf. results) indicates a disadvantage of the constraint *s* = *r* compared to the constraint *λ* = 0.02 we cannot draw definite conclusions about whether subjects perform suboptimal inference, or not, from the present data.

For coherences above about 25% parameter estimates became less reliable (Fig 12A), because accuracy reached its ceiling of 1 and became uninformative. We expect that parameter estimates become more reliable in these experimental conditions, if reaction time distributions are used for fitting instead of only mean reaction times [54].

In the original fits of behaviour in [54] the drift was constrained to be a linear function of coherence ([54], Supp. Fig. 6), where a single parameter, the slope of the linear function replaced coherence-specific drifts. In contrast, in our fits of the BAttM to the same data we allowed both, sensory uncertainty *r* and noise level *s*, to freely vary across coherences. Although this increased flexibility of the BAttM, in principle, could have led to overfitting, it is unlikely that this is the case for our results: The noise in the data is small compared to the effect of the coherence, because the data are averages based on 15,937 trials ([54], Fig 1). The low variance of parameter estimates for intermediate coherences (Fig 12A) also indicates that our fitting method identified unique parameter values for these coherences. Furthermore, by relating the sensory uncertainty parameter in our fits to drift in the drift diffusion model [23], we observed that the fitted values of sensory uncertainty *r* obey the linear constraint employed by [54] for coherences of up to 25% without explicitly using this constraint during fitting. It is currently unclear why the parameters for high coherences do not follow the previously assumed linear relation between drift and coherence. One possible explanation is that the urgency signal, which we did not model in the BAttM, has a larger effect for high coherences than for low ones. The estimated shape of the urgency signal ([54], Supp. Fig. 6b) supports this speculation, because it exhibits a steep rise early in a trial such that its effect should be relatively large for fast decisions. However, clearly further research is required to substantiate this potential mechanism.

### Adapting stimulus expectations

The BAttM explains different behaviour in response to stimuli with different strength using particular combinations of input noise level *s* and sensory uncertainty *r* (Table 2, Fig 12). It, therefore, appears that decision makers adapt their expectations about the stimulus (*r*) to stimulus strength even before they experience the stimulus (we fixed *r* within trials). In experiments in which trials with the same stimulus strength are blocked, or in which stimulus strength is cued before onset of the stimulus, this is plausible. In experiments in which stimulus strength changes randomly across trials, this assumption seems flawed. This consideration has led others to discuss whether the brain implements Bayesian models [72]. Here, we speculate that decision makers rapidly adapt their expectations in parallel with decision making as they sample observations from the stimulus. Such adaptation is compatible with the timescale of short-term synaptic plasticity in the brain [73]. Also, it has previously been demonstrated that sensory reliability (akin to *r*) can be inferred together with stimulus identity in a Bayesian model [25].

Even though we believe that decision makers adapt their stimulus expectations within a trial, the BAttM currently does not employ such a mechanism. Nevertheless, assuming fixed *r* led to good fits of accuracy and mean RTs as recorded in [54] (cf. Fig 12). This is not very surprising: The behavioural data has originally been fit by a drift-diffusion model with constant drift throughout a trial [54]. Such constant drift implements the assumption that the *average* amount of evidence extracted from the stimulus at a given moment is constant throughout the trial. Critically, the ‘evidence’ is not a fundamental, sensory quantity, but needs to be computed by the brain specifically for the given decision problem. It can further be shown [23] that ‘evidence’ depends on sensory uncertainty in probabilistic models. Therefore, the assumption of a constant drift throughout a trial is, in the BAttM, equivalent to maintaining stable expectations about the stimulus throughout the trial. As a result, keeping *r* fixed in the BAttM is a simplification that follows previous approaches based on drift diffusion models and still allows to fit behaviour (accuracy and mean RTs) of subjects well (see Fig 12). Similar to within-trial effects of top-down gain modulation, however, future work may aim at elucidating potential effects of within-trial variations in expected sensory uncertainty *r* due to adaptation of stimulus expectations. In particular, experiments with longer re-decision trials and continuously changing stimulus reliability may induce strong adaptations of stimulus expectations that have measurable behavioural effects.

### Bayesian inference and neurobiological implementation

One of the strengths of the original pure attractor models is their link to possible neurobiological implementations in networks of spiking neurons (cf. Section: pattm). We have abstracted from this perspective and have embedded a pure attractor model in a dynamic Bayesian inference framework. Consequently, the question arises how this apparently more complicated construct may map to a neurobiological substrate. The BAttM is a probabilistic filter that recursively updates posterior beliefs by evaluating the likelihood of the state of a dynamic generative model given a stream of observations (cf. models). A wide range of proposals have been made for how probabilistic filters can be implemented by networks of neurons [47, 74–81]. For example, [80] discusses how computations defined by predictive coding approaches, which derive from probabilistic filters (cf. Section Bayesinf), can map onto canonical microcircuits in cortex. More abstractly, [47, 77, 79] show how networks of rate neurons may implement probabilistic filters and [74–76, 78, 81] provide implementations based on spiking neuron networks. Given these proposals, it seems reasonable to assume that the computations defined by the BAttM can be implemented by the brain.

### Conclusion

We have presented a novel perceptual decision making model, the Bayesian attractor model, which combines attractor dynamics with a probabilistic formulation of decision making. The model captures important behavioural findings and makes novel predictions that can be tested in future experiments. In particular, we have highlighted a re-decision paradigm which can be used to investigate the tradeoff between flexibility and stability in perceptual decisions. Furthermore, the BAttM predicts particular, within-trial modulation of sensory gain which may explain recent experimental findings. Finally, the BAttM predicts experimentally testable links between choice, response times and confidence.

## Methods

### Hopfield dynamics

**Fig 13:**
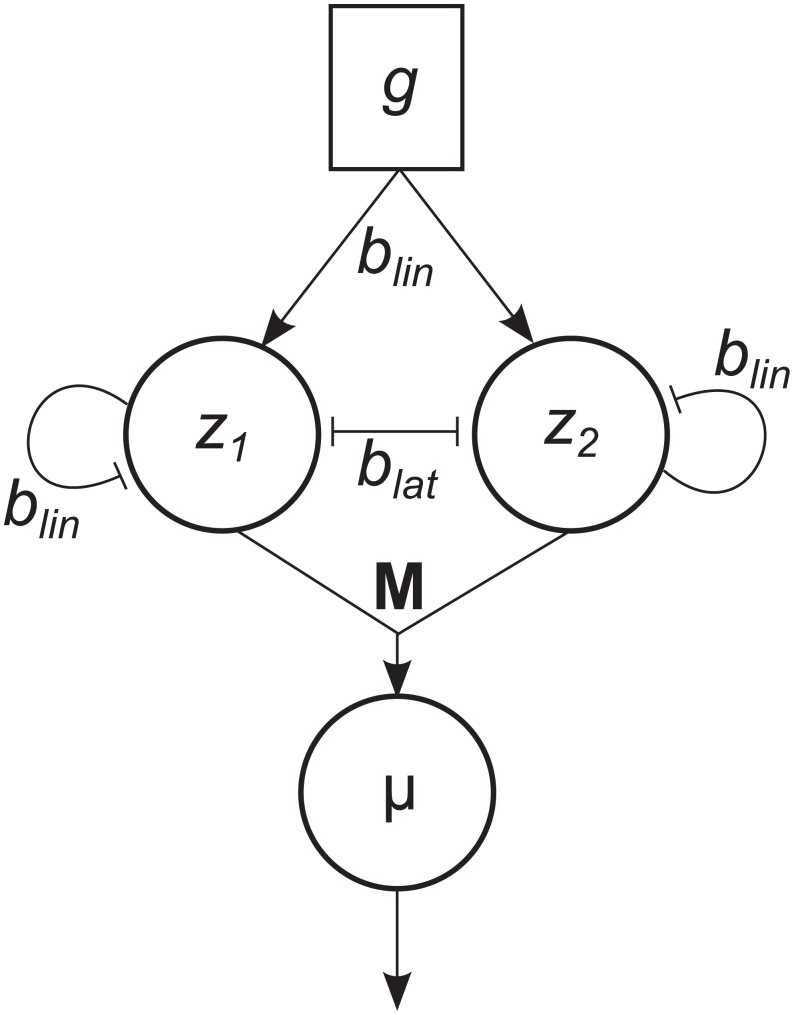
Network diagram for two-alternative Hopfield network (cf. Eqs 9, 10) with interpolated output that was used as generative model. The network is driven by constant input *g* modulated by self and lateral inhibition between state variables *z*_1_ and *z*_2_. The strength of inhibition between state variables is determined by *b* ^*lat*^ (note that self-inhibition is not linear, but moderated by a sigmoid function ***σ***(**z**)) while the strength of self-inhibition and the strength of the constant input is controlled by *b* ^*lin*^. After passing through another sigmoid function ***σ***(**z**) the state variables interpolate target positions (cf. description of single dot task above) stored in **M** and consequently produce the (mean) prediction ***μ***.

We used a Hopfield network as an example of a pure attractor model. Hopfield networks have originally been suggested as a neurobiologically plausible firing-rate models of recurrently connected neurons [44]. We define a general Hopfield network with *N* state variables as follows (here summarised in one equation using matrix notation, see Fig 13 for a graphical representation of the binary case *N* = 2):

$$\dot{z}=k(L\sigma \left(z\right)+b^{lin}\left(g1-z\right))$$ (9)

where **z** ∈ ℝ^*N*^ is the decision state consisting of the state variables *z*_*i*_, *k* is a rate constant, ***σ***(⋅) is a multidimensional logistic sigmoid function and *b* ^*lin*^ is a parameter determining the strength of a goal state attractor **g** = *g* **1**. Lateral inhibition for winner-take-all dynamics is implemented using

$$\sigma_{i}\left(z\right)=\frac{1}{1+e^{-r(z_{i}-o)}}\;\text{and}\;L=b^{lat}\left(I-1\right)$$ (10)

where *r* and *o* determine the slope and centre of the sigmoid function, respectively, *b* ^*lat*^ determines the strength of the lateral inhibition, **1** ∈ ℝ^*N*×*N*^ is a matrix of ones, and **I** is the identity matrix. One can see that the fixed points with one state variable *z*_*m*_ ≈ *g*, while all others are *z*_*j* ≠ *m*_ ≈ 0, are local minima of the underlying Lyapunov function and therefore stable [44] provided that *o* = *g* and *b* ^*lat*^/*b* ^*lin*^ = 2*g*. We denote these stable fixed points as *ϕ*_*m*_ where *m* indicates the state variable that is equal to *g*. As parameter values we used *k* = 4, *g* = 10, *r* = 1, *o* = *g*, *b* ^*lat*^ = 1.7, *b* ^*lin*^ = *b* ^*lat*^/(2*g*) in all experiments, because these provided for numerically stable Hopfield dynamics which exhibited the desired fixed points and reasonably fast convergence to these. For interpolating observations in the generative model (Eq 3) we use the same form of sigmoid as defined in Eq (10), but with parameters *r* = 0.7, *o* = *g*/2. This choice increases the range of values for which the sigmoid is approximately linear and increases robustness of the inference with the generative model.

### Initial decision state

When modelling perceptual decisions, we follow [26, 28] and initialise the attractor dynamics in a neutral state. In particular, we set a prior distribution over the decision state as **z**_0_ ∼ 𝓝(***μ***_0_,**P**_0_) where ***μ***_0_ is an unstable equilibrium point of the Hopfield dynamics for which

$$\mu_{i}=\mu_{j}\;\text{and}\;{\dot{\mu }}_{i}=0\;\forall i,j\in 1,\cdots ,N.$$ (11)

This starting point ensures that a relatively long time is spent close to the equilibrium, while once the dynamics has sufficiently differentiated, the decision state will rapidly move to its closest stable fixed point. We set the covariance of the initial decision state to ${\text{P}}_{0}=p_{0}^{2}\text{I}$ and call *p*_0_ the initial state uncertainty which is a parameter of the model that controls the susceptibility of the decision state to incoming evidence at the beginning of a trial.

### Approximated contour lines

In Fig 6 we plotted contour lines. These were approximated from the noisy data points underlying the grey scale maps as follows. We defined four values for four contours for each map as reported in the caption of Fig 6. For each value, e.g., 500ms, we found all points in the parameter grid for which their own associated value lay within a limit to the chosen contour value (limit of 0.01 fraction correct and of 10ms). We then fitted the hyperparameters of a Gaussian process [82] to the found points in log*r*-log*s* space (one per contour line) using the GPML Matlab toolbox (http://mloss.org/software/view/263/). In particular, the Gaussian process mapped the logarithm of the noise level, log*s*, onto the logarithm of the sensory uncertainty, log*r* and used a standard squared exponential covariance function with a Gaussian likelihood [82]. The contour lines in Fig 6 represent the mean predictions of sensory uncertainty obtained from the fitted Gaussian processes for the corresponding noise level.

### Fitting of data in [54]

To fit the data from the experiment reported in [54] we defined a temporal scaling between our discrete model and the times recorded during the experiment. This scaling corresponds to Δ*t* = 4ms in Eq (2). It was chosen as a tradeoff between sufficiently small discretisation steps and computational efficiency and means that about 200 time steps are sufficient to cover the full range of reaction times observed by [54]. Furthermore, we used a non-decision time of *T*_0_ = 200ms which is roughly the value that was estimated by [54] (cf. their Table 1). The non-decision time captures delays that are thought to be independent of the time that it takes to make a decision. These delays may be due to initial sensory processing, or due to the time that it takes to execute a motor action.

We used a form of stochastic optimisation based on a Markov Chain Monte Carlo (MCMC) method to find parameter values that best explained the observed behaviour in the experiment for each coherence level independently. This was necessary, because we could not analytically predict accuracy and mean reaction times from the model and had to simulate from the model to estimate these quantities. In particular, we simulated 1,000 trials per estimate of accuracy and mean RT, as done to produce Fig 6. We then defined an approximate Gaussian log-likelihood of the parameter set used for simulation by using the estimated values as means:

$$L\left(s,r\right)\propto \frac{{\left(A-\hat{A}\right)}^{2}}{\sigma_{A}^{2}}+\frac{{\left(RT-\hat{RT}\right)}^{2}}{\sigma_{RT}^{2}}+P\left(s,r\right)$$ (12)

where *A* and *RT* are the accuracy and mean RT, respectively, measured in the experiment for one of the coherences and $\hat{A}$ and $\hat{RT}$ are estimates from the model. *σ*_*A*_ = 0.05 and *σ*_*RT*_ = 10ms are ad-hoc estimates of the standard deviation of the estimates which we chose large enough to account for the variability we observed in the data of Fig 6. *P*(*s*,*r*) is a penalty function which returned values greater than 10,000, when more than half of the simulated trials were timed out (cf. light blue areas in Fig 6) and when the particular combination of *s* and *r* lead to too strong overshoots of a state variable (cf. Fig 5A). We identified overshoot parameters as those which lay below a straight line from *r* = 0.47, *s* = 1.45 to *r* = 3.66, *s* = 80 in Fig 6. We embedded the approximate likelihood of Eq (12) into the DRAM method of [83] (Matlab mcmcstat toolbox available at http://helios.fmi.fi/~lainema/mcmc/) which implements adaptive Metropolis-Hastings sampling with delayed rejection. We log-transformed the parameters to ensure that only positive samples are generated and defined wide Gaussian priors in this log-space (log*s* ∼ 𝓝(0,10^2^), log*r* ∼ 𝓝(0,10^2^)), but also constrained *s* > 0.1 to ensure a minimum level of noise. We then ran the MCMC method for 3,000 samples, discarded the first 499 samples and chose every 5th sample to reduce correlations within the Markov chain. The resulting set of 501 parameter samples is a rough approximation of the posterior distribution over parameters for the given data. It is not statistically exact, because of the approximate likelihood, but it still indicates when parameter estimates become unreliable, as demonstrated in Fig 12. The parameter values reported in Table 2 are those of the sample (of the 501) which fitted the behaviour for a given coherence best, as determined by Eq (12).

Note that, different from [54], we did not a priori assume a particular relationship between coherence and the parameters of the BAttM during fitting. In [54] coherence linearly scaled the drift in their drift-diffusion model using a scaling parameter *K* that was common across coherences ([54], Supp. Fig. 6), that is, the average amount of momentary evidence accumulated in the model was determined from the coherence used in a trial. In the BAttM the fitted parameters, sensory uncertainty *r* and noise level *s*, determine how stimulus features are translated into momentary evidence. Since we did not want to assume, a priori, a specific relationship between the level of coherence and parameters *s* and *r*, we chose to let the parameters vary independently of coherence during fitting. However, we investigated whether an equivalent relation between *r* and coherence holds for the fitted values of *r*. As stated in the main text, this relation can be written as *r* ^2^ = *K*′/*c* where *c* is coherence and *K*′ is an arbitrary constant. Consequently, we used a least-squares approach to estimate *K*′ from given pairs of coherence (in %) and sensory uncertainty *r* (Table 2). The best fitting value was *K*′ = 381.9. As suggested by one reviewer, it may be useful to assume the above relation between *r* ^2^ and *c* as a constraint when fitting noisy data. This can be easily done by fitting *K*′ to the data across coherences instead of directly fitting one *r* per coherence.
